# MRI-based prediction of microvascular invasion/high tumor grade and adjuvant therapy benefit for solitary HCC ≤ 5 cm: a multicenter cohort study

**DOI:** 10.1007/s00330-024-11295-1

**Published:** 2024-12-19

**Authors:** Hanyu Jiang, Binrong Li, Tianying Zheng, Yun Qin, Yuanan Wu, Zhenru Wu, Maxime Ronot, Victoria Chernyak, Kathryn J. Fowler, Mustafa R. Bashir, Weixia Chen, Yuan-Cheng Wang, Shenghong Ju, Bin Song

**Affiliations:** 1 Department of Radiology, Functional and Molecular Imaging Key Laboratory of Sichuan Province, West China Hospital, Sichuan University, Chengdu, Sichuan China; 2 Jiangsu Key Laboratory of Molecular and Functional Imaging, Department of Radiology, Zhongda Hospital, School of Medicine, Southeast University, Nanjing, Jiangsu China; 3 Laboratory of Pathology, Key Laboratory of Transplant Engineering and Immunology, NHC, West China Hospital, Sichuan University, Chengdu, Sichuan China; 4 Université Paris Cité, UMR 1149, CRI, Paris & Service de Radiologie, Hôpital Beaujon, APHP.Nord, Clichy, France; 5 Department of Radiology, Memorial Sloan Kettering Cancer Center, NYC, New York, NY USA; 6 Department of Radiology, University of California San Diego, San Diego, CA USA; 7 Department of Radiology, Center for Advanced Magnetic Resonance in Medicine, and Division of Gastroenterology, Department of Medicine, Duke University Medical Center, Durham, NC USA; 8 Department of Radiology, Sanya People’s Hospital, Sanya, Hainan China

**Keywords:** Carcinoma, Hepatocellular, Magnetic resonance imaging, Prognosis

## Abstract

**Objectives:**

To develop and externally validate an MRI-based diagnostic model for microvascular invasion (MVI) or Edmondson-Steiner G3/4 (i.e., high-risk histopathology) in solitary BCLC 0/A hepatocellular carcinoma (HCC) ≤ 5 cm and to assess its performance in predicting adjuvant therapy benefits.

**Materials and methods:**

This multicenter retrospective cohort study included 577 consecutive adult patients who underwent contrast-enhanced MRI and subsequent curative resection or ablation for solitary BCLC 0/A HCC ≤ 5 cm (December 2011 to January 2024) from four hospitals. For resection-treated patients, a diagnostic model integrating clinical and 50 semantic MRI features was developed against pathology with logistic regression analyses on the training set (center 1) and externally validated on the testing dataset (centers 2–4), with its utilities in predicting posttreatment recurrence-free survival (RFS) and adjuvant therapy benefit evaluated by Cox regression analyses.

**Results:**

Serum α-fetoprotein > 100 ng/mL (odds ratio (OR), 1.94; *p* = 0.006), non-simple nodular growth subtype (OR, 1.69; *p* = 0.03), and the VICT2 trait (OR, 4.49; *p* < 0.001) were included in the MVI or high-grade (MHG) trait, with testing set AUC, sensitivity, and specificity of 0.832, 74.0%, and 82.5%, respectively. In the multivariable Cox analysis, the MHG-positive status was associated with worse RFS (resection testing set HR, 3.55, *p* = 0.02; ablation HR, 3.45, *p* < 0.001), and adjuvant therapy was associated with improved RFS only for the MHG-positive patients (resection HR, 0.39, *p* < 0.001; ablation HR, 0.30, *p* = 0.005).

**Conclusion:**

The MHG trait effectively predicted high-risk histopathology, RFS and adjuvant therapy benefit among patients receiving curative resection or ablation for solitary BCLC 0/A HCC ≤ 5 cm.

**Key Points:**

***Question*** *Despite being associated with increased recurrence and potential benefit from adjuvancy in HCC, microvascular invasion or Edmondson-Steiner grade 3/4 are hardly assessable noninvasively.*

***Findings*** *We developed and externally validated an MRI-based model for predicting high-risk histopathology, post-resection/ablation recurrence-free survival, and adjuvant therapy benefit in solitary HCC ≤ 5 cm.*

***Clinical relevance*** *Among patients receiving curative-intent resection or ablation for solitary HCC ≤ 5 cm, noninvasive identification of high-risk histopathology (MVI or high-grade) using our proposed MRI model may help improve individualized prognostication and patient selection for adjuvant therapies.*

## Introduction

Microvascular invasion (MVI) and Edmondson-Steiner G3/4 are established adverse prognostic factors in hepatocellular carcinoma (HCC) [1–4]. While there are currently no standardized adjuvant therapies for HCC after curative treatment, growing evidence from clinical trials has suggested a role for adjuvant therapies in improving oncological outcomes for patients at high risk for recurrence [5–8], as recommended in the Chinese guidelines [1, 4]. Most of these trials defined the high-risk factors as tumor multiplicity, large tumor size (e.g., > 5 cm), macrovascular invasion, MVI, and Edmondson-Steiner G3/4 [5–7].

While tumor number, size, and macrovascular invasion are readily evaluable on pretreatment imaging, the MVI and Edmondson-Steiner grade assessments still mandate pathological analyses. Thus, these data are typically not available when determining the use of adjuvant therapy after ablation since pretreatment biopsy is neither required for HCC diagnosis nor reliable for MVI assessment [7, 9]. Therefore, noninvasive identification of patients with high-risk histopathology (i.e., presence of MVI or Edmondson-Steiner G3/4) but without other high-risk factors (i.e., solitary Barcelona Clinic Liver Cancer (BCLC) 0/A HCC ≤ 5 cm) may help improve patient selection for adjuvant therapies, particularly in those treated with ablation.

MRI has been widely acknowledged as the most effective imaging modality to evaluate HCC aggressiveness [10, 11]. MRI features, such as hepatobiliary phase (HBP) peritumoral hypointensity and its non-hepatobiliary-specific alternative VICT2 trait (i.e., based on peritumoral portal venous phase hypoenhancement, incomplete “capsule,” corona enhancement and peritumoral mild-moderate T2 hyperintensity) have been associated with the presence of MVI [10, 12–16]; while nonperipheral washout and marked HBP hypointensity are  indicative of Edmondson-Steiner G3/4 tumors [10, 17, 18]. Despite encouraging results, the performance of MRI in predicting high-risk histopathology and adjuvant therapy benefit for patients receiving curative treatment for BCLC 0/A HCC ≤ 5 cm remains unclear.

Therefore, we aimed to develop and externally validate an MRI-based diagnostic model for high-risk histopathology (i.e., presence of MVI or Edmondson-Steiner G3/4) in the resection-treated patients with solitary BCLC 0/A HCC ≤ 5 cm, and to investigate the model’s utilities in predicting recurrence-free survival (RFS) and adjuvant therapy benefit following curative resection or radiofrequency ablation (RFA).

## Materials and methods

This multicenter retrospective cohort study was approved by the institutional review boards at four tertiary-care referral hospitals in China, including West China Hospital of Sichuan University (center 1), Zhongda Hospital of Southeast University, Sanya People’s Hospital, and Chengdu ShangJin NanFu Hospital (centers 2–4), with waived requirements to obtain informed consent.

### Patients

**Fig. 1:**
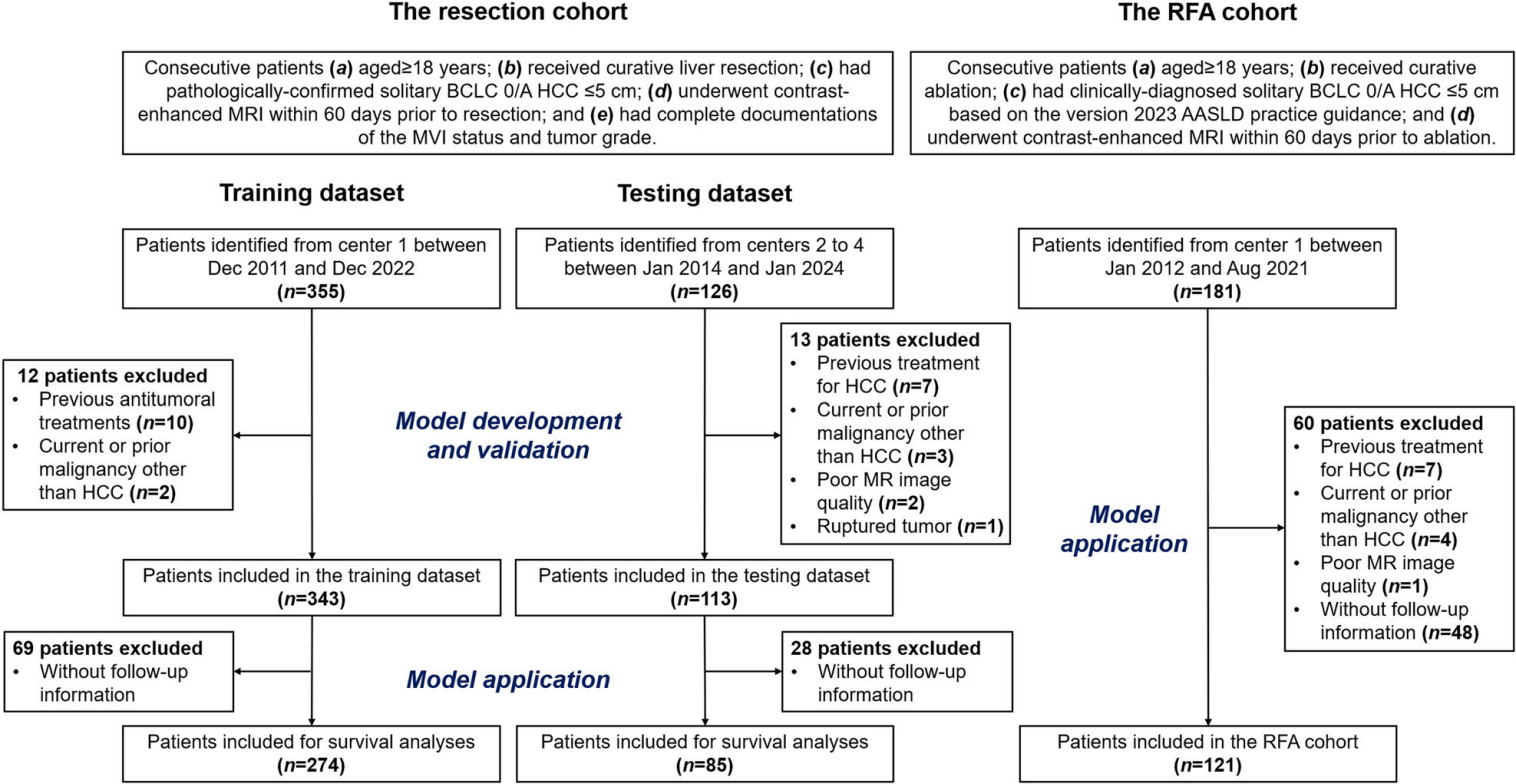
Study flowchart. BCLC, Barcelona Clinic Liver Cancer; HCC, hepatocellular carcinoma; MVI, microvascular invasion; AASLD, American Association for the Study of Liver Diseases; RFA, radiofrequency ablation

Two cohorts, namely “the resection cohort” (collected from four centers) and “the RFA cohort” (collected exclusively from center 1), comprised the study population (Fig. 1).

For the resection cohort, between December 2011 and January 2024, consecutive patients were identified if: (1) aged ≥ 18 years; (2) received curative liver resection; (3) had pathologically confirmed solitary BCLC 0/A HCC ≤ 5 cm; (4) underwent contrast-enhanced MRI within 60 days prior to resection [19]; and (5) had complete documentations of the MVI status and tumor grade. Patients were excluded if they had: (1) any previous antitumoral treatments; (2) any current or prior malignancy other than HCC; (3) poor MR image quality (e.g., severe artifacts); or (4) a ruptured tumor.

For the RFA cohort, patients were included following similar criteria. Specifically, between January 2012 and August 2021, consecutive patients were identified if: (1) aged ≥ 18 years; (2) received curative RFA (4); (3) had clinically diagnosed solitary BCLC 0/A HCC ≤ 5 cm based on the criteria of the American Association for the Study of Liver Diseases (AASLD) [1]; and (4) underwent contrast-enhanced MRI within 60 days prior to RFA. Patients were excluded if they had: (1) any previous antitumoral treatments, (2) any current or prior malignancy other than HCC, (3) poor MR image quality, (4) a ruptured tumor, or (5) no follow-up information.

Patient demographics, underlying chronic liver diseases, and laboratory results within 14 days before resection/RFA were recorded. Adjuvant therapies were suggested for patients with anticipated high risk of recurrence (e.g., presence of MVI for resection and elevated posttreatment serum tumor markers for RFA) according to the perceived probabilities of success based on multidisciplinary team discussions and patient preferences [4–7]. Frequently used adjuvant regimens, alone or in combination, included transarterial chemoembolization, systemic therapy, and radiotherapy, with the selection tailored in an individualized manner (illustrated in Supplementary Fig. 1).

### MRI acquisition and analysis

The MRIs were acquired using various 1.5-T or 3.0-T MR scanners. Either extracellular or hepatobiliary contrast agents were used based on radiologist recommendations or multidisciplinary team discussions. MR acquisition parameters are detailed in Supplementary Material 1 and Supplementary Table 1.

The MRIs were centrally reviewed by three independent fellowship-trained abdominal radiologists who had 3 to 10 years of experience in liver MRI. All reviewers were aware of the HCC diagnosis but blinded to the remaining clinical, pathological, and follow-up information.

Fifty MRI features were evaluated, including (1) the Liver Imaging Reporting and Data System v2018 features [20]; (2) other previously reported prognostic imaging features, such as tumor growth subtype (simple nodular type or non-simple nodular type) and the VICT2 trait [10–16, 21–27]; and (3) imaging features associated with the severity of the underlying liver disease (e.g., radiologically evident cirrhosis) [28]. Definitions of the tumor-related prognostic imaging features not included in the Liver Imaging Reporting and Data System v2018 are summarized in Table 1, while definitions of all evaluated imaging features are detailed in Supplementary Table 2.

**Table 1:** Tumor-related prognostic MRI features and definitions not included in the version 2018 Liver Imaging Reporting and Data System

Imaging features	Definition	Image sequence
Pre-contrast T1-weighted hypointensity (present vs. absent)	Signal intensity of the tumor on pre-contrast T1-weighted imaging unequivocally lower than that of the liver.	Pre-contrast T1WI
T2-weighted peritumoral hyperintensity (present vs. absent)	Presence of irregular, wedge-shaped, or flame-like area adjacent to the tumor on T2WI of which the signal intensity is mildly or moderately higher than liver and similar to or less than a non-iron-overloaded spleen [15, 16].	T2WI
Portal venous phase peritumoral hypoenhancement (present vs. absent)	Presence of irregular, wedge-shaped, or flame-like hypoenhancing area adjacent to the tumor in the portal venous phase [15, 16].	Portal venous phase
Arterial phase hyperenhancement proportion (< 50% vs. ≥ 50%)	Proportion of tumor volume demonstrating arterial phase hyperenhancement [21].	Late arterial phase
Intratumoral artery (present vs. absent)	Presence of discrete arteries within the tumor on arterial phase images [15, 22, 23].	Early or late arterial phase
Capsule integrity (complete vs. incomplete)	Tumor capsule is complete when non-disrupted capsule is detected in all imaging planes, otherwise incomplete [15].	Multiple sequences
Tumor margin (nonsmooth vs. smooth)	Nonsmooth: the tumor margin is irregular and/or has areas of bulging, nodular projection, or infiltration into adjacent tissues at the tumor periphery in any imaging plane. Smooth: the tumor margin is smooth in all imaging planes [15, 23].	Multiple sequences
Marked hepatobiliary phase hypointensity (present vs. absent)	Signal intensity of the tumor in the hepatobiliary phase lower than that of the liver and similar to or lower than that of intrahepatic vessels [24].	Hepatobiliary phase
Hepatobiliary phase peritumoral hypointensity (present vs. absent)	Presence of irregular, wedge-shaped or flame-like hypointense area of liver parenchyma located outside of the tumor margin in the hepatobiliary phase [12, 15, 16, 24].	Hepatobiliary phase
The VICT2 trait (present vs. absent)	The VICT2 trait is present when portal venous phase peritumoral hypoenhancement is present; or when corona enhancement, T2-weighted peritumoral mild-moderate hyperintensity, and incomplete capsule are all present; otherwise, absent [16].	Multiple sequences
The two-trait predictor of venous invasion (present vs. absent)	Presence of intratumoral artery coupled with absence of nonenhancing “capsule” [22, 23].	Multiple sequences
Tumor growth subtype [25, 26]		Multiple sequences
Simple nodular type	A round expanding nodule with a distinct margin in all imaging planes.	
Non-simple nodular type	An expanding tumor with areas of bulging or nodular extranodular projection, a cluster of small and confluent nodules, or the presence of an indistinct margin throughout the entire tumor.	

Disagreements were resolved with the majority interpretations for binary imaging features, and by consulting a senior abdominal radiologist with over 20 years of experience in liver MRI for categorical imaging features when necessary.

### Reference standard

Histopathologic data from routine reports were retrieved as the reference standard to determine the MVI status and Edmondson-Steiner grade. In compliance with the institutional standard procedures, two liver pathologists who were aware of the clinical and imaging data reviewed all specimens in consensus at each center. For heterogenous tumors with more than one Edmondson-Steiner grade identified, the highest grade was documented for being more prognostic. High-risk histopathology was defined as the presence of MVI or Edmondson-Steiner G3/4.

### Patient follow-up

Patients were regularly followed up after treatment at 1 month, every 3 months for the first 2 years, and every 3–6 months afterward with serum AFP and contrast-enhanced imaging techniques (ultrasound, CT, or MRI) until death or the last available follow-up date. Bone scintigraphy or biopsies were performed as clinically indicated. Recurrence was defined as unequivocal radiological or pathologic identification of intrahepatic HCC, tumor-in-vein, or distant metastasis [1]. RFS was defined as the time from treatment to first-documented tumor recurrence or all-cause death.

### Statistical analysis

Inter-rater agreement was assessed with the Fleiss’ κ value for binary imaging features, intraclass correlation coefficient for continuous imaging features, and weighted κ value for categorical imaging features.

No formal sample size calculation was performed beforehand, but the large number of events compared to the number of variables analyzed at the multivariable regression analyses guaranteed the “ten events per variable” rule, thus implying sufficient accuracy of the regression estimates [29].

For the resection cohort, the data of patients from center 1 comprised the training set for developing a diagnostic model (i.e., the **M**VI or **h**igh-**g**rade (MHG) model) to predict high-risk histopathology, while the data of those from centers 2 to 4 comprised the testing set for validating the MHG model. For the RFA cohort, the data of patients (collected exclusively from center 1) was used to assess the MHG’s utilities in predicting posttreatment RFS and informing adjuvant therapy benefits.

#### Development and validation of the MHG model in the resection cohort

In the training set of the resection cohort, uni- and multivariable logistic regression analyses were performed to identify clinical and MRI predictors for high-risk histopathology while controlling for patient age and sex. Laboratory parameters were assessed as continuous variables and dichotomized according to ranges of normality or clinical relevance (cutoffs detailed in Supplementary Table 3). For variables that were collinear according to Spearman’s correlation analysis or the variance inflation factors, the variable with the smallest *p*-value at univariable analysis was selected. All independent variables with univariable *p* < 0.05 were analyzed in the multivariable logistic regression model using the backward stepwise method and five-fold cross-validation. The MHG model was formulated as a score based on the variables from the multivariable logistic analysis weighted by their respective β coefficients, with the largest β coefficient scaled as 20 points and the remaining proportionally rounded to the nearest integers to improve clinical utility. The optimal threshold score of the MHG model was determined by receiver operating characteristic analysis with Youden’s index.

In the testing set, model discrimination was evaluated with the area under the receiver operating characteristic curve (AUC), sensitivity, specificity, positive predictive value (PPV), negative predictive value (NPV), and accuracy. Model calibration was assessed by the calibration curve. Subgroup analyses were performed for tumors ≤ 2 cm and 2–5 cm based on domain knowledge.

#### Survival analyses

Survival outcomes were estimated by the Kaplan–Meier method and compared with the log-rank test. The MHG’s utilities to predict posttreatment RFS and adjuvant therapy benefit were compared with high-risk histopathology for the resection cohort, and with the IMBRAVE 050 trial-defined high-risk status (i.e., single tumor > 2 cm but ≤ 5 cm) for the RFA cohort since this trial is currently the largest international phase III randomized control trial for the adjuvant therapy of HCC [7]. To adjust for the impacts of other prognostic factors, uni- and multivariable Cox regression analyses were performed for established clinical, radiological, and treatment-associated prognostic factors not included in the model. The Cox proportional hazards assumption was verified by the smoothed plots of Schoenfeld residuals.

The statistical analyses were conducted using Medcalc (version 20.112; MedCalc Software) or R (version 4.2.2; R Foundation for Statistical Computing). A two-tailed *p* < 0.05 was considered statistically significant.

## Results

### Patients

Patient characteristics are summarized in Table 2.

**Table 2:** Baseline patient characteristics

Characteristics	The resection cohort				The RFA cohort	*p*-value^b^
	All	Train	Test	*p*-value^a^		
No. patients	456	343	113	…	121	…
High-risk histopathology^c^	188 (41.2)	138 (40.2)	50 (44.2)	0.45	…	…
Age, years	55 (47–64)	54 (47–63)	56 (49–66)	0.052	59 (51–68)	**0.003**
Sex, male	382 (83.8)	285 (83.1)	97 (85.8)	0.49	99 (81.8)	0.61
Chronic liver disease^d^						
Hepatitis B	400 (87.7)	298 (86.9)	102 (90.3)	0.34	98 (81.0)	0.06
Hepatitis C	24 (5.3)	17 (5.0)	7 (6.2)	0.61	7 (5.8)	0.82
Alcohol abuse	5 (1.1)	3 (0.9)	2 (1.8)	0.60	23 (19.0)	**< 0.001**
Others	3 (0.7)	1 (0.3)	2 (1.8)	0.15	0 (0)	> 0.99
Cryptogenic	29 (6.4)	29 (8.5)	0 (0)	**0.001**	11 (9.1)	0.31
Cirrhosis	293 (64.3)	224 (65.3)	69 (61.1)	0.41	91 (75.2)	**0.02**
Child-Pugh class				0.11		**< 0.001**
A	448 (98.2)	339 (98.8)	109 (96.5)		106 (87.6)	
B	8 (1.8)	4 (1.2)	4 (3.5)		15 (12.4)	
Tumor size, cm	2.8 (2.0–3.8)	2.7 (2.0–3.5)	3.2 (2.1–4.3)	**< 0.001**	2.1 (1.6–2.9)	**< 0.001**
α-fetoprotein, ng/mL				0.63		0.13
≤ 100	292 (64.0)	219 (63.8)	75 (66.4)		87 (71.9)	
> 100	162 (35.5)	124 (36.2)	38 (33.6)		34 (28.1)	
Aspartate transaminase, U/L				0.34		**< 0.001**
≤ 35	323 (70.8)	247 (72.0)	76 (67.3)		63 (52.1)	
> 35	133 (29.2)	96 (28.0)	37 (32.7)		58 (47.9)	
Alanine transaminase, U/L				0.60		0.79
≤ 40	326 (71.5)	243 (70.8)	83 (73.5)		85 (70.2)	
> 40	130 (28.5)	100 (29.2)	30 (26.5)		36 (29.8)	
Albumin, g/L				**< 0.001**		**< 0.001**
≥ 40	352 (77.2)	279 (81.3)	73 (64.6)		66 (54.5)	
< 40	104 (22.8)	64 (18.7)	40 (35.4)		55 (45.5)	
Total bilirubin, µmol/L				0.15		**0.04**
≤ 40	454 (99.6)	342 (99.7)	111 (98.2)		117 (96.7)	
> 40	3 (0.7)	1 (0.3)	2 (1.8)		4 (3.3)	
Platelet count, × 10^9^/L				**0.002**		**< 0.001**
≥ 100	297 (65.1)	210 (61.2)	87 (77.0)		40 (33.1)	
< 100	159 (34.9)	133 (38.8)	26 (23.0)		81 (66.9)	
Microvascular invasion	150 (32.9)	113 (32.9)	37 (32.7)	0.97	…	…
Edmondson-Steiner grade				0.87		…
I-II	369 (80.9)	277 (80.8)	92 (81.4)		…	…
III-IV	87 (19.1)	66 (19.2)	21 (18.6)		…	…
Median follow-up time^e^, months	27.2 (19.5–39.9)	29.3 (21.0–40.3)	23.6 (15.2–38.0)	**0.01**	43.0 (25.8–67.8)	**< 0.001**

Unless stated otherwise, data in parentheses are percentages or interquartile ranges. *p*-values < 0.05 are highlighted in bold

*RFA* radiofrequency ablation

^a^ *p*-values were computed by comparing the training (*n* = 343) and testing (*n* = 113) centers

^b^ *p*-values were computed by comparing the resection (*n* = 456) and RFA (*n* = 121) cohorts

^c^ The high-risk histopathology was defined as the presence of microvascular invasion or Edmondson-Steiner G3/4 tumors at postoperative pathological examinations

^d^ More than one etiology could be present for each patient. Metabolic dysfunction associated fatty liver disease, hepatitis A, and autoimmune hepatitis were grouped within the category “others”

^e^ Data are presented for the resection cohort patients who had follow-up data (training center, *n* = 273; testing center, *n* = 64), and for all the RFA cohort patients (*n* = 121)

Briefly, 577 patients (median age, 55 years; IQR, 48–64 years; 481 (83.4%) males) were included, 456 (79.0%) (training set, *n* = 343; testing set, *n* = 113) and 121 (21.0%) from the resection and RFA cohorts, respectively. Among them, 498 (86.3%) had hepatitis B virus (HBV) infection, and 384 (66.6%) had cirrhosis.

For the resection cohort, MVI and Edmondson-Steiner G3/4 tumors were respectively observed in 150 (32.9%) and 87 (19.1%) patients; thus, 188 (41.2%) patients had high-risk histopathology. Compared with the testing set, the training set patients had smaller tumors (median size, 2.7 cm vs. 3.2 cm, *p* < 0.001), less frequent albumin reduction (18.7% vs. 35.4%, *p* < 0.001), and more frequent platelet reduction (38.8% vs. 23.0%, *p* = 0.002).

Compared with the resection cohort, the RFA cohort patients were older in age (median age, 59 vs. 55 years, *p* = 0.003), had smaller tumors (median size, 2.1 cm vs. 2.8 cm, *p* < 0.001), more chronic liver disease secondary to alcohol abuse (19.0% vs. 1.1%, *p* < 0.001), more frequent cirrhosis (75.2% vs. 64.3%, *p* = 0.02), Child-Pugh B liver function (12.4% vs. 1.8%, *p* < 0.001) and elevations in aspartate transaminase (47.9% vs. 29.2%, *p* < 0.001) as well as total bilirubin (3.3% vs. 0.7%, *p* = 0.04), and more frequent reductions in albumin (45.5% vs. 22.8%, *p* < 0.001) as well as platelet count (66.9% vs. 34.9%, *p* < 0.001).

### Development of the MHG model in the training set of the resection cohort

In the multivariable logistic analysis, the VICT2 trait (odds ratio (OR), 4.49; 95% CI, 2.39–8.44; *p* < 0.001; corresponding to 20 points for its presence), serum AFP (OR, 1.94; 95% CI, 1.21–3.11; *p* = 0.006; corresponding to 9 points for > 100 ng/mL), and non-simple nodular growth subtype (OR, 1.69; 95% CI, 1.06–2.70; *p* = 0.03; corresponding to 7 points for its presence) were associated with high-risk histopathology (Table 3). Thus, the MHG model was formulated as below:

**Table 3:** Training center uni- and multivariable logistic regression analyses based on majority interpretations

Predictors	Univariable analysis		Multivariable analysis			
	Odds ratio	*p*-value	β	Odds ratio	*p*-value	Score points
The Barcelona Clinic Liver Cancer stage (A vs. 0)	1.79 (1.09–2.94)	0.02	…	…	…	…
Serum α-fetoprotein (> 100 ng/mL vs. ≤ 100 ng/mL)	1.98 (1.27–3.11)	0.003	0.66	1.94 (1.21–3.11)	0.006	9 points for > 100 ng/mL
Tumor size (cm)	1.31 (1.06–1.62)	0.01	…	…	…	…
Peritumoral mild-moderate T2 hypointensity (present vs. absent)	3.79 (1.93–7.46)	< 0.001	…	…	…	…
Corona enhancement (present vs. absent)	2.23 (1.39–3.57)	< 0.001	…	…	…	…
Portal venous phase peritumoral hypoenhancement (present vs. absent)	3.71 (1.75–7.86)	< 0.001	…	…	…	…
Capsule integrity (complete vs. incomplete)	0.50 (0.31–0.79)	0.003	…	…	…	…
Tumor margin (nonsmooth vs. smooth)	1.83 (1.15–2.91)	0.01	…	…	…	…
Tumor growth subtype (non-simple nodular type vs. simple nodular type)	2.11 (1.36–3.28)	< 0.001	0.53	1.69 (1.06–2.70)	0.03	7 points for the non-simple nodular type
Necrosis or severe ischemia (present vs. absent)	2.39 (1.33–4.30)	0.004	…	…	…	…
The VICT2 trait (present vs. absent)^a^	5.18 (2.81–9.55)	< 0.001	1.50	4.49 (2.39–8.44)	< 0.001	20 points for its presence

Unless stated otherwise, data in parentheses are 95% confidence intervals

The the **M**VI or **H**igh-**g**rade (MHG) score = 20 × the VICT2 trait (present, 1; absent, 0) + 9 × serum α-fetoprotein (> 100 ng/mL, 1; ≤ 100 ng/mL, 0) + 7 × tumor growth subtype (the non-simple nodular type, 1; the simple nodular type, 0)

The optimal threshold of the MHG score for predicting high-risk histopathology was > 9 points

^a^ The VICT2 trait is an integrated MR feature based on peritumoral mild-moderate T2 hyperintensity, corona enhancement, portal venous phase peritumoral hypoenhancement, and capsule integrity

The MHG model = 20 × the VICT2 trait (present, 1; absent, 0) + 9 × serum AFP (> 100 ng/mL, 1; ≤ 100 ng/mL, 0) + 7 × tumor growth subtype (non-simple nodular type, 1; simple nodular type, 0).

The optimal threshold score of the MHG model was > 9 points. Therefore, the MHG model was simplified as a decision-making algorithm (i.e., the MHG trait), which corresponded as positive when the VICT2 trait was present or when serum AFP > 100 ng/mL and non-simple nodular type were both present; otherwise, negative.

**Fig. 2:**
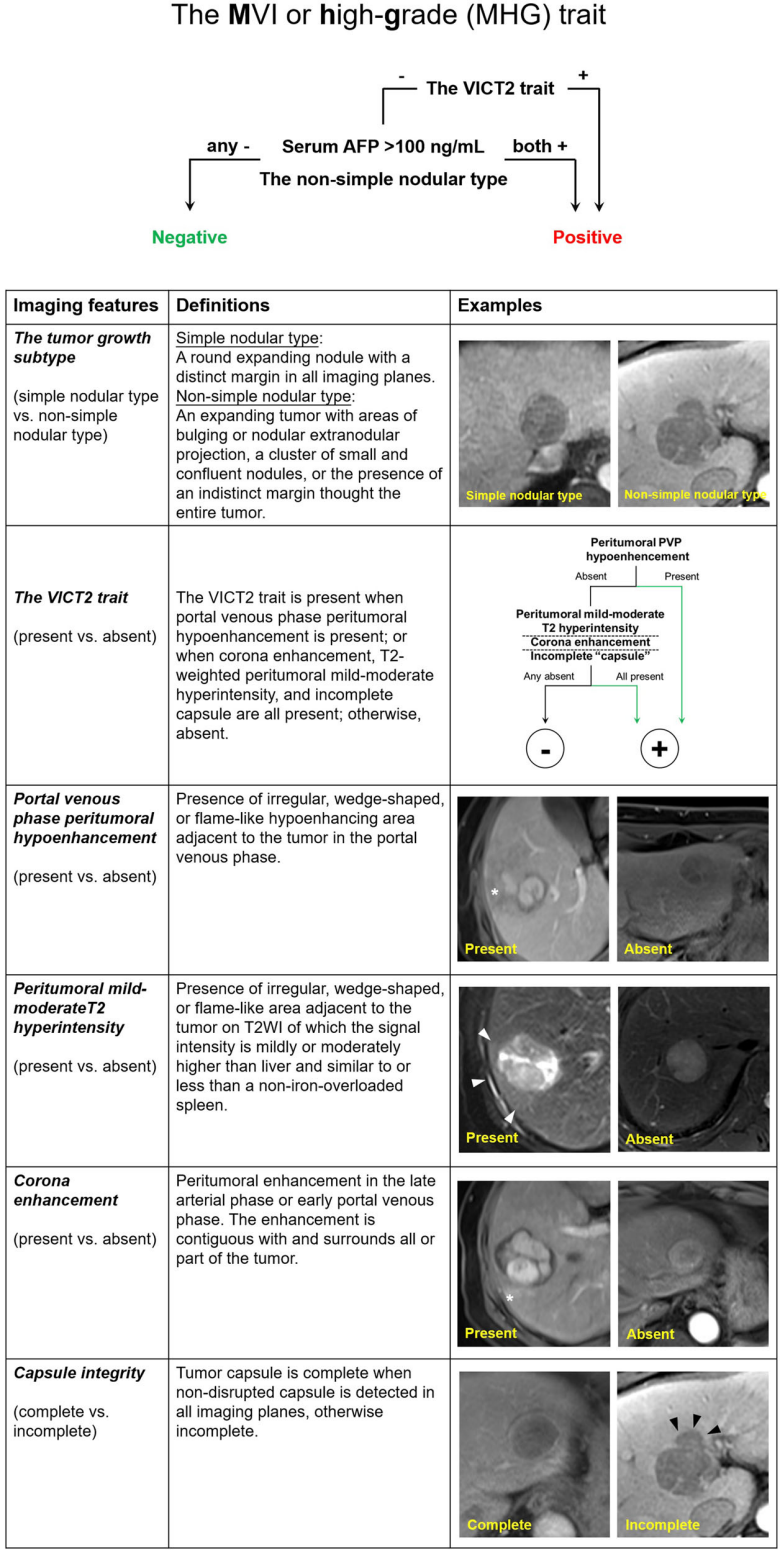
Graphical illustration of the MVI or high-grade (MHG) model. AFP, α-fetoprotein

The MHG trait is graphically illustrated in Fig. 2, and the MRIs of a typical MHG-positive patient are shown in Supplementary Fig. 2. Inter-rater agreements for the imaging features are summarized in Supplementary Table 4.

### Validation of the MHG model in the testing set of the resection cohort

Testing set diagnostic estimates based on majority interpretations are summarized in Table 4.

**Table 4:** Testing dataset diagnostic estimates of the MHG trait based on majority interpretations

	All	≤ 2 cm	> 2 cm, ≤ 5 cm
AUC	0.832 (0.750–0.896)	0.809 (0.603–0.937)	0.834 (0.740–0.905)
True positive	37	6	31
False negative	13	2	11
False positive	11	3	8
True negative	52	14	38
Sensitivity	74.0% (59.7–85.4%)	75.0% (34.9–96.8%)	73.8% (58.0–86.1%)
Specificity	82.5% (70.9–90.9%)	82.4% (56.6–96.2%)	82.6% (68.6–92.2%)
PPV	77.1% (65.7–85.5%)	66.7% (39.9–85.8%)	79.5% (66.8–88.2%)
NPV	80.0% (71.2–86.6%)	87.5% (67.4–96.0%)	77.6% (67.1–85.4%)
Accuracy	78.8% (70.1–86.0%)	80.0% (59.3–93.2%)	78.4% (68.4–86.5%)

Unless stated otherwise, data in parentheses are 95% confidence intervals

*AUC* area under the receiver operating characteristic curve, *PPV* positive predictive value, *NPV* negative predictive value

Briefly, the AUC of the MHG model was 0.832 (95% CI, 0.750–0.896) for the entire population (*n* = 113), 0.809 (95% CI, 0.603–0.937) for patients with tumors ≤ 2 cm (*n* = 25), and 0.834 (95% CI, 0.740–0.905) for those with tumors 2–5 cm (*n* = 88) (Supplementary Fig. 3). The sensitivity, specificity, PPV, NPV, and accuracy of the MHG trait were 74.0% (95% CI, 59.7–85.4%), 82.5% (95% CI, 70.9–90.9%), 77.1% (95% CI, 65.7–85.5%), 80.0% (95% CI, 71.2–86.6%), and 78.8% (95% CI, 70.1–86.0%) for the entire population; 75.0% (95% CI, 34.9–96.8%), 82.4% (95% CI, 56.6–96.2%), 66.7% (95% CI, 39.9–85.8%), 87.5% (95% CI, 67.4–96.0%), and 80.0% (95% CI, 59.3–93.2%) for patients with tumors ≤ 2 cm; and 73.8% (95% CI, 58.0–86.1%), 82.6% (95% CI, 68.6–92.2%), 79.5% (95% CI, 66.8–88.2%), 77.6% (95% CI, 67.1–85.4%), and 78.4% (95% CI, 68.4–86.5%) for those with tumors 2–5 cm, respectively.

The calibration curves showed that the MHG score was well-calibrated (Supplementary Fig. 3).

### Prediction of posttreatment RFS

#### The resection cohort

For the resection cohort, 274 (79.9%) training and 85 (75.2%) testing set patients had follow-up information. The median follow-up time was 29.3 months (IQR, 21.0–40.3 months) for the training set and 23.6 months (IQR, 15.2–38.0 months) for the testing set (*p* = 0.01). During the follow-up period, 32.1% (88/274) of training set patients and 21.2% (18/85) of testing set patients experienced recurrence (*p* = 0.055), while 6.9% (19/274) of training set patients and 5.9% (5/85) of testing set patients died (*p* = 0.93).

**Fig. 3:**
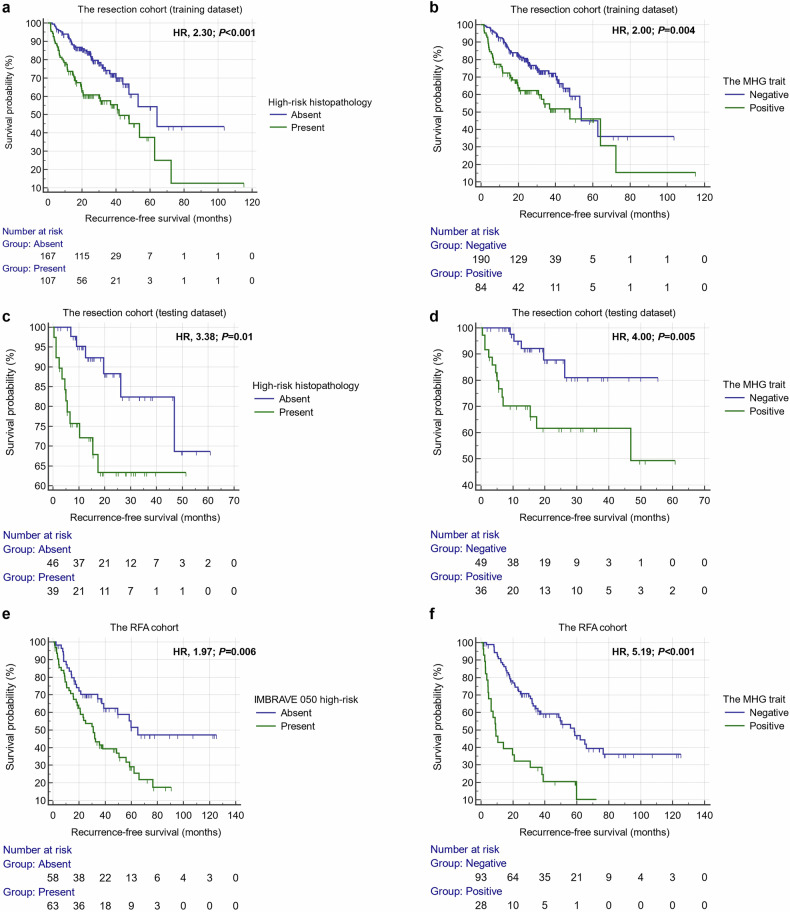
Recurrence-free survival outcomes stratified by high-risk histopathology (**a**, **c**), the IMRAVE 050-defined high-risk status (i.e., single tumor > 2 cm but ≤ 5 cm (**e**), and the MHG trait (**b**, **d**, **f**). MHG, MVI or high-grade; HR, hazard ratio; RFA, radiofrequency ablation

The training set RFS was worse for patients with high-risk histopathology (median RFS, 41.8 vs. 64.1 months; hazard ratio (HR), 2.30; 95% CI, 1.49–3.55; *p* < 0.001), and for those with the MHG-positive status (median RFS, 47.7 vs. 53.8 months; HR, 2.00; 95% CI, 1.25–3.19; *p* = 0.004). Similarly, the testing set RFS was worse for patients with high-risk histopathology (median RFS, not reached for either group; HR, 3.38; 95% CI, 1.29–8.81; *p* = 0.01), and for those with the MHG-positive status (median RFS, 46.9 months vs. not reached; HR, 4.00; 95% CI, 1.53–10.42; *p* = 0.005) (Fig. 3). The MHG-positive status remained an independent risk factor for worse RFS while adjusting for other established prognostic factors for both the training (HR, 1.61; 95% CI, 1.03–2.51; *p* = 0.04) and testing (HR, 3.55; 95% CI, 1.25–10.10; *p* = 0.02) sets in the multivariable Cox regression analyses (Supplementary Table 5).

#### The RFA cohort

For the RFA cohort, during a median follow-up time of 43.0 months (IQR, 25.8–67.8 months), 66 (54.5%) patients experienced recurrence, and 26 (21.5%) patients died. RFS was worse for patients with the MHG-positive status (median RFS, 9.1 vs. 58.4 months; HR, 5.19; 95% CI, 2.61–10.33; *p* < 0.001), as well as for those with the IMBRAVE 050-defined high-risk status (median RFS, 30.9 vs. 65.0 months; HR, 1.97; 95% CI, 1.21–3.19; *p* = 0.006) (Fig. 3). The MHG-positive status remained an independent risk factor for RFS (HR, 3.45; 95% CI, 2.01–5.80; *p* < 0.001) when adjusting for other established prognostic factors in the multivariable Cox regression analysis (Supplementary Table 5).

### Prediction of adjuvant therapy benefit

#### The resection cohort

Adjuvant therapy regimens are detailed in Supplementary Table 6. Briefly, 27.9% (100/359) of the resection cohort patients who had follow-up information received adjuvant therapies.

**Fig. 4:**
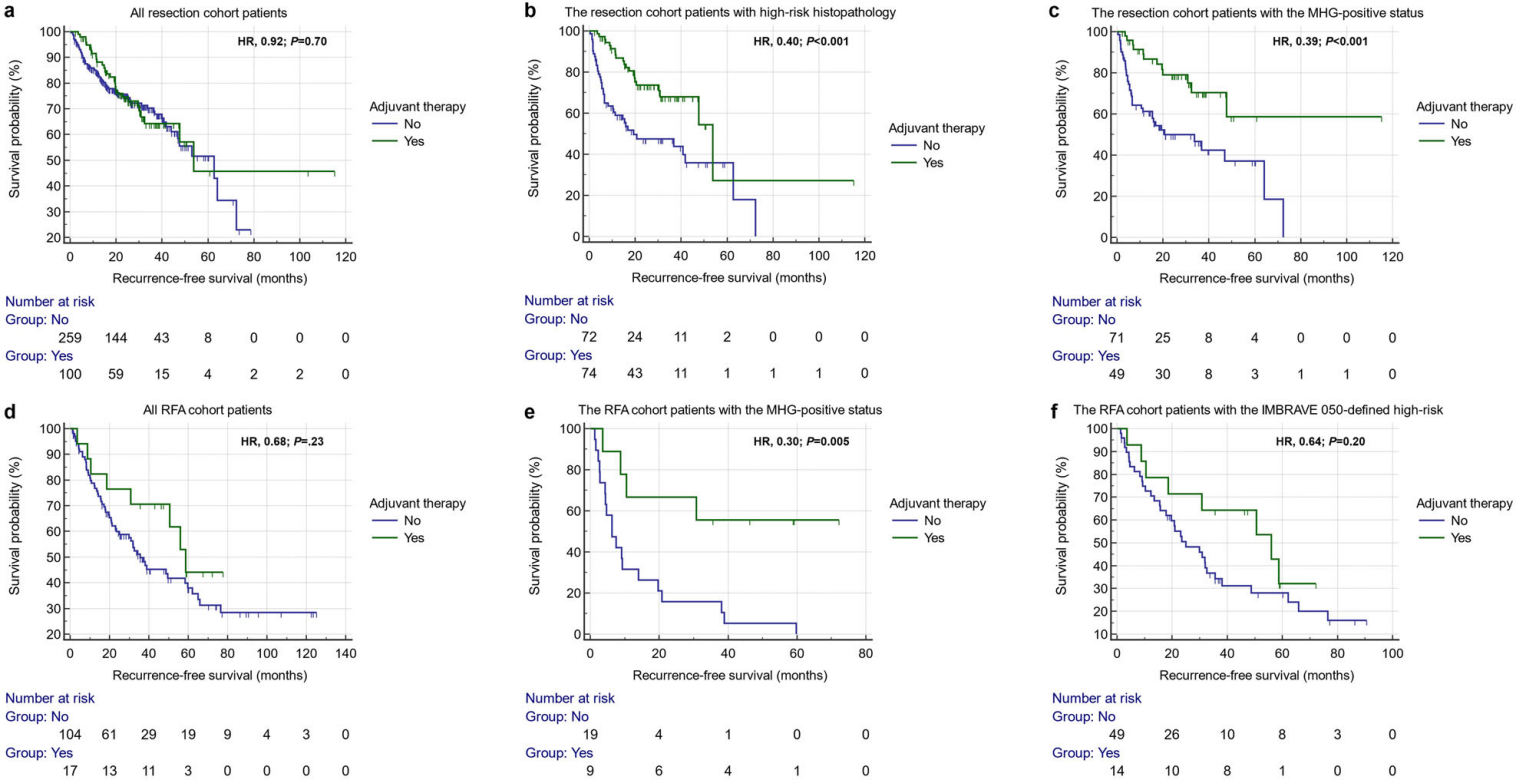
Recurrence-free survival outcomes between patients with and without adjuvant therapies for the resection (**a**–**c**) and RFA (**d**–**f**) cohorts. The IMBRAVE 050-defined high-risk status was single tumor > 2 cm but ≤ 5 cm (7). MHG, MVI or high-grade; HR, hazard ratio; RFA, radiofrequency ablation

For the entire resection cohort patients (*n* = 359), RFS outcomes were similar for those with and without adjuvant therapies (median RFS, 53.8 vs. 62.7 months; HR, 0.92; 95% CI, 0.60–1.40; *p* = 0.70). However, after risk stratification based on pathology, the use of adjuvant therapies was associated with improved RFS (median RFS, 53.8 vs. 19.3 months; HR, 0.40; 95% CI, 0.24–0.66; *p* < 0.001) for patients with high-risk histopathology (*n* = 146), but not for those with low-risk histopathology (HR, 1.54; 95% CI, 0.64–3.69; *p* = 0.33) (*n* = 213) (Fig. 4). Furthermore, for patients with high-risk histopathology, the use of adjuvant therapies remained a protective factor for improved RFS while adjusting for other established prognostic factors in the multivariable Cox regression analysis (HR, 0.39; 95% CI, 0.23–0.68; *p* < 0.001) (Table 5).

**Table 5:** Uni- and multivariable Cox regression analyses of adjuvant therapy administration and known prognostic factors for recurrence-free survival in patients with high-risk histopathology or the MHG-positive status

Characteristics	Univariable Cox analysis		Multivariable Cox analysis^a^	
	Hazard ratio	*p*-value	Hazard ratio	*p*-value
The resection cohort, patients with high-risk histopathology (*n* = 146)				
Adjuvant therapy (yes vs. no)	0.40 (0.23–0.67)	**< 0.001**	0.40 (0.23–0.68)	**< 0.001**
Age (years)	0.99 (0.97–1.02)	0.58	...	...
Sex (male vs. female)	1.05 (0.50–2.23)	0.89	...	...
Chronic hepatitis B virus infection (yes vs. no)	0.77 (0.24–2.48)	0.67		
Cirrhosis (present vs. absent)	0.98 (0.59–1.63)	0.93	...	...
Tumor size (cm)	1.32 (1.07–1.63)	**0.01**	1.31 (1.06–1.61)	**0.01**
The Barcelona Clinic Liver Cancer stage (A vs. 0)	2.08 (0.99–4.39)	0.054	...	...
The albumin-bilirubin grade (2 vs. 1)	1.24 (0.69–2.24)	0.47	...	...
Aspartate aminotransferase (IU/L)	1.00 (0.99–1.01)	0.92	...	...
Alanine aminotransferase (IU/L)	1.00 (0.99–1.01)	0.60	...	...
Total bilirubin (µmol/L)	1.02 (0.98–1.06)	0.36	...	...
Albumin (g/L)	1.02 (0.96–1.08)	0.51	...	...
Platelet count (10^9^/L)	1.00 (0.99–1.00)	0.51	...	...
Prothrombin time (s)	0.95 (0.75–1.19)	0.65	...	...
The resection cohort, patients with the MHG-positive status (*n* = 120)				
Adjuvant therapy (yes vs. no)	0.35 (0.18–0.67)	**0.001**	0.37 (0.19–0.72)	**0.003**
Age (years)	0.99 (0.97–1.02)	0.53	...	...
Sex (male vs. female)	1.57 (0.56–4.39)	0.39	...	...
Chronic hepatitis B virus infection (yes vs. no)	1.14 (0.45–2.90)	0.78	...	...
Cirrhosis (present vs. absent)	1.03 (0.58–1.81)	0.93	...	...
Tumor size (cm)	1.24 (0.97–1.58)	0.08	...	...
The Barcelona Clinic Liver Cancer stage (A vs. 0)	2.62 (0.81–8.45)	0.11	...	...
The albumin-bilirubin grade (2 vs. 1)	1.93 (1.04–3.59)	**0.04**	...	...
Aspartate aminotransferase (IU/L)	1.02 (1.01–1.04)	**< 0.001**	...	...
Alanine aminotransferase (IU/L)	1.02 (1.01–1.03)	**0.002**	1.01 (1.00–1.03)	**0.009**
Total bilirubin (µmol/L)	1.02 (0.97–1.06)	0.45	...	...
Albumin (g/L)	0.96 (0.91–1.02)	0.20	...	...
Platelet count (10^9^/L)	1.00 (1.00–1.01)	0.47	...	...
Prothrombin time (s)	0.95 (0.75–1.19)	0.64	...	...
The RFA cohort, patients with the MHG-positive status (*n* = 28)				
Adjuvant therapy (yes vs. no)	0.23 (0.08–0.69)	**0.009**	0.23 (0.08–0.69)	**0.009**
Age (years)	0.99 (0.96–1.03)	0.80	...	...
Sex (male vs. female)	2.55 (0.58–11.11)	0.21	...	...
Chronic hepatitis B virus infection (yes vs. no)	0.41 (0.14–1.15)	0.09	...	...
Cirrhosis (present vs. absent)	1.42 (0.52–3.85)	0.49	...	...
Tumor size (cm)	1.02 (0.64–1.63)	0.93	...	...
The Barcelona Clinic Liver Cancer stage (A vs. 0)	0.90 (0.35–2.32)	0.83	...	...
The albumin-bilirubin grade (2/3 vs. 1)	1.38 (0.58–3.32)	0.47	...	...
Aspartate aminotransferase (IU/L)	1.02 (1.00–1.04)	0.10	...	...
Alanine aminotransferase (IU/L)	1.02 (1.00–1.04)	0.12	...	...
Total bilirubin (µmol/L)	1.03 (0.99–1.08)	0.18	...	...
Albumin (g/L)	1.01 (0.91–1.13)	0.78	...	...
Platelet count (10^9^/L)	1.00 (0.99–1.00)	0.56	...	...
Prothrombin time (s)	0.91 (0.62–1.32)	0.60	...	...

Unless stated otherwise, data in parentheses are 95% confidence intervals. *p*-values < 0.05 are highlighted in bold

*MHG* the microvascular invasion or high-grade trait, *RFA* radiofrequency ablation

^a^ To avoid overfitting, variables with *p* < 0.05 at the univariable Cox regression analyses were analyzed in the multivariable Cox regression models

Similarly, after risk stratification based on the MHG trait, the use of adjuvant therapies was associated with improved RFS (median RFS, not reached vs. 20.7 months; HR, 0.39; 95% CI, 0.22–0.67; *p* < 0.001) for patients with the MHG-positive status (*n* = 120), but not for those with the MHG-negative status (HR, 1.92; 95% CI, 1.00–3.70; *p* = 0.052) (*n* = 239) (Fig. 4). Furthermore, for patients with the MHG-positive status, the use of adjuvant therapies remained an independent protective factor for longer RFS while adjusting for other established prognostic factors in the multivariable Cox regression analysis (HR, 0.37; 95% CI, 0.19–0.72; *p* = 0.003) (Table 5).

#### The RFA cohort

Among patients from the RFA cohort, 17 (14.0%) received adjuvant therapies (regimens detailed in Supplementary Table 6). For the entire RFA cohort, RFS were similar for patients with and without adjuvant therapies (median RFS, 58.7 vs. 38.6 months; HR, 0.68; 95% CI, 0.36–1.27; *p* = 0.23). After risk stratification based on the MHG trait, the use of adjuvant therapies was associated with improved RFS (median RFS, not reached vs. 6.3 months; HR, 0.30; 95% CI, 0.13–0.69; *p* = 0.005) for the MHG-positive patients (*n* = 28), but not for the MHG-negative ones (HR, 0.76; 95% CI, 0.30–1.90; *p* = 0.56) (*n* = 93) (Fig. 4). Furthermore, for patients with the MHG-positive status, the use of adjuvant therapies remained a protective factor for improved RFS while adjusting for other established prognostic factors (HR, 0.23; 95% CI, 0.08–0.69; *p* = 0.009) in the multivariable Cox regression analysis (Table 5). However, for patients with the IMBRAVE 050-defined high-risk status (*n* = 63), no difference in RFS was detected between patients with and without adjuvant therapies (HR, 0.64; 95% CI, 0.32–1.26; *p* = 0.20) (Fig. 4).

## Discussion

Noninvasive assessment of high-risk histopathology is critical for individualized prognostication and adjuvant therapy administration in HCC. In 577 patients with solitary BCLC 0/A HCCs ≤ 5 cm from four tertiary-care referral hospitals, we developed and externally validated a noninvasive MHG trait to diagnose high-risk histopathology (i.e., the presence of MVI or Edmondson-Steiner G3/4) based on serum AFP and two MRI features, which showed a testing set AUC of 0.832. For both the resection and RFA cohorts, the MHG trait could effectively predict posttreatment RFS independent of several established prognostic factors. Furthermore, for both cohorts, the use of adjuvant therapies was associated with improved RFS only for the MHG-positive patients (Kaplan–Meier analysis: resection, HR, 0.40, *p* < 0.001; ablation HR, 0.30, *p* = 0.005), rather than the entire patient population (Kaplan–Meier analysis: resection, HR, 0.92, *p* = 0.70; ablation, HR, 0.68, *p* = 0.23).

The MHG trait may serve as a noninvasive diagnostic tool for high-risk histopathology in HCC, and its performance was strong for tumors ≤ 2 cm (testing set AUC, 0.809) and those between 2 and 5 cm (testing set AUC, 0.834). These results aligned with previous works, in which contrast-enhanced MRI was reported effective in separately evaluating the MVI status and tumor grade [12–14, 16, 18]. Noteworthily, the MHG trait was developed exclusively on patients with solitary BCLC 0/A HCCs ≤ 5 cm, because noninvasive assessment of high-risk histopathology is less relevant and reliable for more advanced-stage tumors. Specifically, more advanced-stage tumors generally carry an elevated risk for MVI, higher tumor grade, and worse RFS; thus, noninvasive assessment of high-risk histopathology is less relevant for treatment decision-making [12–18]. Furthermore, considering the marked histological heterogeneity within and between tumors, sampling errors may bias the results in large or multifocal tumors, making the pathological examinations less reliable [12–14, 18].

In the current study, serum AFP and two contrast-enhanced MRI features constituted the MHG trait. All these features are readily measurable and thus may be easily applied in routine practice. Among them, the non-simple nodular tumor growth subtype represents an imaging analog to the non-simple nodular gross pathologic subtype proposed by the Liver Cancer Study Group of Japan [30], which has been associated with higher tumor grade, more frequent MVI, reduced capsule formation, and increased expression of stemness-related markers compared to the simple nodular subtype [25, 26, 31]. Furthermore, the VICT2 trait, a recently described non-hepatobiliary-specific imaging alternative to HBP peritumoral hypointensity, has been linked to increased MVI and worse postoperative RFS in HCC [16].

In addition to diagnosing high-risk histopathology, the MHG trait also showed promise as a noninvasive prognostic and decision-making tool, as it allowed effective prediction of posttreatment RFS and adjuvant therapy benefit, independent of several established prognostic factors. These findings are clinically relevant. First, for patients awaiting resection, those with MHG-positive status may benefit from more aggressive resection procedures, such as a wider resection margin, anatomic resection, and major resection [32–35]. Second, for patients eligible for both resection and ablation (e.g., adequate performance status, preserved liver function, absence of clinically significant portal hypertension and single small tumors), those with the MHG-positive status might benefit more from resection over ablation [14]. Finally, for both the resection and RFA cohorts, the use of adjuvant therapies was associated with improved RFS only for the MHG-positive patients rather than the entire population. Despite the critical utility of high-risk histopathology as an indicator for the use of adjuvant therapies [5–9], the MVI status and Edmondson-Steiner grade are not typically evaluated for RFA-treated HCC. This, in part, explained why an exclusively size-based criterion (i.e., > 2 cm but ≤ 5 cm) was adopted by the IMBRAVE 050 trial to define the high-risk status for solitary HCC treated with ablation. However, the use of adjuvant therapies was not associated with improved RFS for the IMBRAVE 050-defined high-risk patients in our work, which echoed the negative results for ablation-treated patients in IMBRAVE 050 (unadjusted HR, 0.61; 95% CI, 0.26–1.41) [7]. By contrast, the MHG trait was more effective for predicting adjuvant therapy benefit in RFA-treated patients, supported by the results that adjuvant therapy was associated with improved RFS for the MHG-positive patients but not for the IMBRAVE 050-defined high-risk ones. These results revealed a current paucity of effective decision-making tool to inform the use of adjuvant therapies for ablation-treated solitary BCLC 0/A HCC and highlight the potential of the MHG trait to meet such needs. Furthermore, this work also stood as a representative among few studies that extrapolated the findings from resection to ablation [14, 36]. Nevertheless, these potential clinical implications were derived from single-center retrospective data, and any reliable conclusion would require intensive external prospective validations, ideally in the settings of clinical trials.

This study had several limitations. First, as a retrospective study, selection bias might have impacted our results. Second, up to 86.3% of the included patients had HBV infection. Therefore, our model may not perform as well in non-HBV populations. Third, the RFA cohort comprised of limited number of patients and was only collected from the training set. Therefore, further external prospective validations are needed to testify the applicability of MHG trait for ablation-treated patients. Finally, adjuvant therapies were only documented for the training set and were recommended based on multidisciplinary discussions in an individualized but non-standardized manner, which might hurdle the extrapolations of our findings. Therefore, future large-scale multicenter prospective studies evaluating HCC patients with more diverse chronic liver disease etiologies are warranted to test and refine our findings.

In conclusion, based on 577 patients from four tertiary-care referral hospitals, we developed and externally validated an MRI-based MHG trait that could effectively predict high-risk histopathology, posttreatment RFS and adjuvant therapy benefit for patients undergoing curative resection or RFA for solitary BCLC 0/A HCC ≤ 5 cm.

## Supplementary information

Supplementary Material

## Abbreviations

AFP: Alpha-fetoprotein
AUC: Area under the receiver operating characteristic curve
BCLC: Barcelona Clinic Liver Cancer
HBP: Hepatobiliary phase
HBV: Hepatitis B virus
HCC: Hepatocellular carcinoma
HR: Hazard ratio
IQR: Interquartile range
MVI: Microvascular invasion
NPV: Negative predictive value
PPV: Positive predictive value
RFS: Recurrence-free survival
