# Supplementary material for: MRI-based prediction of microvascular invasion/high tumor grade and adjuvant therapy benefit for solitary HCC ≤ 5 cm: a multicenter cohort study
Source: Eur Radiol. 2024 Dec 19;35(6):3223–37. doi: 10.1007/s00330-024-11295-1 (PMC12081510; doi:10.1007/s00330-024-11295-1)
Supplement: Supplementary file 1 — Supplementary Material [file 330_2024_11295_MOESM1_ESM.pdf]

Supplemental Material 1: Imaging acquisition details

Training dataset

For the training dataset, all contrast-enhanced MR images were acquired on one of the following eight types of 3.0 T or 1.5 T MR systems (Siemens MAGNETOM Skyra 3.0 T; Siemens TrioTim 3.0 T; Siemens Avanto 1.5 T; GE SIGNA™ Architect 3.0 T; GE SIGNA™ Premier 3.0 T; GE Discovery MR 750 3.0 T; Philips Ingenia Elition X 3.0 T; uMR588 1.5 T) using either extracellular contrast-enhanced MRI (ECA-MRI) or gadoxetate disodium-enhanced MRI (EOB-MRI). For the acquisition of ECA-MRI, 0.1 mmol/kg of gadopentetate dimeglumine (Magnevist®; Bayer Schering Pharma AG), gadoterate meglumine (Dotarem®; Guerbet), or gadobenate dimeglumine (MultiHance®; Bracco) was injected at a rate of 2.5 ml/s. For the acquisition of EOB-MRI, 0.025 mmol/kg of EOB (Primovist®; Bayer Schering Pharma AG or XianAi®; Chia Tai TianQing Pharmaceutical Co., Ltd) was injected at a rate of 1.0-2.0 ml/s.

All patients were asked to fast for 6-8 hours before MR examinations. The MRI sequences included: (***a***) T2-weighted imaging; (***b***) diffusion-weighted imaging (b values: 0, 50, 500, 800, 1000, and 1200 s/mm^2 [Siemens MAGNETOM Skyra]; 0, 50, 800 s/mm^2 [Siemens TrioTim; Siemens Avanto]; 0, 50, 1000 s/mm^2 [Siemens Avanto; GE SIGNA™ Architect; GE SIGNA™ Premier; uMR588]; 0, 200, 800, and 1000 s/mm^2 [GE Discovery MR 750]; 0, 200, 1000 s/mm^2 [Philips Ingenia Elition X]) with apparent diffusion coefficient maps reconstructed using the monoexponential model; (***c***) in- and out-of-phase T1-weighted imaging; and (***d***) dynamic T1-weighted imaging in the pre-contrast phase, late arterial phase, portal venous phase (60 s after the start of contrast media injection), delayed phase (for ECA-MRI, 3min after start of contrast media injection) or transitional phase (for EOB-MRI, 5min after the start of contrast media injection), and hepatobiliary phase (for EOB-MRI, 20min after the start of contrast media injection). The arterial phase images were acquired with either a bolus-tracking method (acquisition triggered 7s after the arrival of the contrast bolus in the celiac trunk) or a multiple arterial phase imaging technique (acquired with an 18 s-breath-hold 20s after the start of contrast media injection and further reconstructed with a temporal resolution of 3 s).

Testing dataset

For the testing dataset, seven types of MRI scanners (GE Signa HDxt 1.5 T, GE Discovery MR 750w 3.0 T, Philips Achieva 3.0 T, Philips Ingenia 3.0 T, Siemens Verio 3.0 T, Siemens MAGNETOM Vida 3.0 T, uMR780 3.0 T) were used for the acquisition of contrast-enhanced MR images. Both ECA and EOB were used. For the acquisition of ECA-MRI, 0.1 mmol/kg of gadopentetate dimeglumine (Magnevist®; Bayer Schering Pharma AG or SCHERING) or gadoteric acid meglumine (JiaDiXian®; HENGRUI) was injected at a rate of 2.5 ml/s. For the acquisition of EOB-MRI, 0.025 mmol/kg of EOB (Primovist®; Bayer Schering Pharma AG or XianAi®; Chia Tai TianQing Pharmaceutical Co., Ltd) was injected at a rate of 1.0-2.0 ml/s.

The MRI sequences included (***a***) T2-weighted imaging; (***b***) diffusion-weighted imaging (b values: 0, and 600s/mm^2 [GE Signa HDxt 1.5 T, Siemens Verio 3.0 T]; 0, and 800 s/mm^2 [Philips Achieva 3.0 T, Philips Ingenia 3.0 T]; 0, and 1000 s/mm^2 [GE Discovery MR 750w 3.0 T]; 0, 50, and 800s/mm^2 [Siemens MAGNETOM Vida 3.0 T]; 0, 50, and 1000s/mm^2 [uMR 780 3.0 T] with apparent diffusion coefficient maps reconstructed using the monoexponential model; (***c***) in- and out-of-phase T1-weighted imaging; and (***d***) dynamic T1-weighted imaging in the pre-contrast phase, late arterial phase, portal venous phase (60s after the start of contrast media injection), delayed phase (for ECA-MRI, 3min after start of contrast media injection) or transitional phase (for EOB-MRI, 5min after the start of contrast media injection), and hepatobiliary phase (for EOB-MRI, 20min after the start of contrast media injection).

Detailed acquisition parameters for the training and testing centers are summarized in **Supplemental Table 1**.

Supplemental Table 1. MR sequences and parameters.

Sequence	Fat suppression	TR (ms)	TE (ms)	Flip angle (°)	ST (mm)	Spacing (mm)	Matrix size	FOV (mm^2)	Acquisition Time (s)
**Training dataset: Siemens MAGNETOM Skyra 3.0 T (18-channel body array coil)**									
T2-weighted 2D FSE	Yes	2160	100	160	6	1.8	320×288	433×433	36
Diffusion-weighted imaging*	Yes	5600	68	90	6	1.8	100×76	380×289	233
In- and out-of-phase T1-weighted imaging	No	81	2.72/1.4	70	6	1.8	352×286	400×325	24
Dynamic T1-weighted 3D GRE	Yes	3.95	1.92	9	2.5	-	352×256	400×296	14
**Training dataset: Siemens TrioTim 3.0 T (8-channel body anterior coil)**									
T2-weighted 2D FSE	Yes	2700	95	140	6	7.8	320×147	442×254	Respiratory gating
Diffusion-weighted imaging*	Yes	5900	76	90	6	7.8	192×154	393×393	245
In- and out-of-phase T1-weighted imaging	No	181	2.2/3.67	65	6	7.8	256×131	410×269	18
Dynamic T1-weighted 3D GRE	Yes	3.47	1.25	9	2.4	-	320×133	434×257	17

Note. —TR = repetition time; TE = echo time; ST = section thickness; FOV = field of view; 2D = two-dimensional; 3D = three-dimensional; FSE = fast spin-echo; GRE = gradient recall echo. * Images were acquired under free breath.

Sequence	Fat suppression	TR (ms)	TE (ms)	Flip angle (°)	ST (mm)	Spacing (mm)	Matrix size	FOV (mm^2)	Acquisition Time (s)
**Training dataset: Siemens Avanto 1.5 T (30-channel body anterior coil)**									
T2-weighted 2D FSE	Yes	2530	84	150	6	7.8	256×187	293×251	47
Diffusion-weighted imaging*	Yes	3600	88	90	6	7.8	192×115	310×232	92
In- and out-of-phase T1-weighted imaging	No	72	4.92/2.22	70	6	7.8	256×158	328×225	16
Dynamic T1-weighted 3D GRE	Yes	5.41	2.39	10	2.5	-	320×138	382×238	15
**Training dataset: Siemens Avanto 1.5 T (8-channel body anterior coil)**									
T2-weighted 2D FSE	Yes	2710	84	150	7.5	9.75	256×177	308×380	27
Diffusion-weighted imaging*	Yes	2000	72	90	7.5	9.75	192×125	308×379	20
In- and out-of-phase T1-weighted imaging	No	87	4.92/2.22	70	7.5	9.75	256×187	308×380	33
Dynamic T1-weighted 3D GRE	Yes	5.4	2.38	10	2	-	320×131	241×407	15

Note. —TR = repetition time; TE = echo time; ST = section thickness; FOV = field of view; 2D = two-dimensional; 3D = three-dimensional; FSE = fast spin-echo; GRE = gradient recall echo. * Images were acquired under free breath.

Sequence	Fat suppression	TR (ms)	TE (ms)	Flip angle (°)	ST (mm)	Spacing (mm)	Matrix size	FOV (mm^2)	Acquisition Time (s)
**Training dataset: GE SIGNA™ Architect 3.0 T (30-channel body anterior coil)**									
T2-weighted 2D FSE	Yes	2400	85	111	7	2	320×192	380×304	34
Diffusion-weighted imaging*	Yes	5000	Minimum	90	7	2	160×128	380×342	Respiratory gating
In- and out-of-phase T1-weighted imaging	No	233.8	2.3/1.1	55	7	2	160×288	380×323	18
Dynamic T1-weighted 3D GRE	Yes	3.9	1.7	15	3	-	320×240	380×380	15
**Training dataset: GE SIGNA™ Premier 3.0 T (30-channel body anterior coil)**									
T2-weighted 2D FSE	Yes	2200	85	111	7	2	320×224	304×380	47
Diffusion-weighted imaging*	Yes	5000	Minimum	90	7	2	120 × 240	380× 380	Respiratory gating
In- and out-of-phase T1-weighted imaging	No	146.8	2.3/1.1	55	7	2	320×192	342×380	16
Dynamic T1-weighted 3D GRE	Yes	3.2	1.4	15	2.4	-	320×240	380× 380	15

Note. —TR = repetition time; TE = echo time; ST = section thickness; FOV = field of view; 2D = two-dimensional; 3D = three-dimensional; FSE = fast spin-echo; GRE = gradient recall echo. * Images were acquired under free breath.

Sequence	Fat suppression	TR (ms)	TE (ms)	Flip angle (°)	ST (mm)	Spacing (mm)	Matrix size	FOV (mm^2)	Acquisition Time (s)
**Training dataset: GE Discovery MR 750 3.0 T (16-channel phased-array torsor coil)**									
T2-weighted 2D FSE	Yes	6315	78	111	6	2	288×244	360×280	Respiratory gating
Diffusion-weighted imaging*	Yes	9230	Minimum	90	6	2	128 × 128	360× 380	Respiratory gating
In- and out-of-phase T1-weighted imaging	No	150	2.5/1.3	70	6	2	288×192	420×420	31
Dynamic T1-weighted 3D GRE	Yes	4.1	1.9	15	2	-	512×512	380× 300	15
**Training dataset: Philips Ingenia Elition X 3.0 T (16-channel body anterior coil)**									
T2-weighted 2D FSE	Yes	1883.51	90	90	6.8	8.5	272×78	239×69	46
Diffusion-weighted imaging*	Yes	1653.65	60.29	90	7	8.5	142×140	141×139	52
In- and out-of-phase T1-weighted imaging	No	164.53	2.30/1.15	50	6	7.5	256×201	206×162	11
Dynamic T1-weighted 3D GRE	Yes	4.20	0.00	10	3	1.5	344×252	303×222	13

Note. —TR = repetition time; TE = echo time; ST = section thickness; FOV = field of view; 2D = two-dimensional; 3D = three-dimensional; FSE = fast spin-echo; GRE = gradient recall echo. * Images were acquired under free breath.

Sequence	Fat suppression	TR (ms)	TE (ms)	Flip angle (°)	ST (mm)	Spacing (mm)	Matrix size	FOV (mm^2)	Acquisition Time (s)
**Training dataset: uMR588 1.5 T (6-channel body anterior coil)**									
T2-weighted 2D FSE	Yes	2600	99.2	90	6.5	1.5	256×168	427×320	39
Diffusion-weighted imaging*	Yes	3350	77	90	6.5	10	128×92	320×400	Respiratory gating
In- and out-of-phase T1-weighted imaging	No	117.6	4.69/2.26	60	6.5	1.3	256×174	320×400	29
Dynamic T1-weighted 3D GRE	Yes	4.2	1.88	10	2.5	-	256×154	255×400	13

Note. —TR = repetition time; TE = echo time; ST = section thickness; FOV = field of view; 2D = two-dimensional; 3D = three-dimensional; FSE = fast spin-echo; GRE = gradient recall echo. * Images were acquired under free breath.

Sequence	Fat suppression	TR (ms)	TE (ms)	Flip angle (°)	ST (mm)	Spacing (mm)	Matrix size	FOV (mm^2)	Acquisition Time (s)
**Testing dataset: GE Signa HDxt 1.5 T (8-channel body upper coil)**									
T2-weighted 2D FSE	Yes	6666.7	86	90	6	7	288×224	390×390	192
Diffusion-weighted imaging*	Yes	6000.0	68.5	90	6	7	128×128	390×390	98
In- and out-of-phase T1-weighted imaging	No	180	4.7/2.2	90	6	7	288×160	390×390	22
Dynamic T1-weighted 3D GRE	Yes	3.9	1.8	15	5.4	2.7	288×200	390×390	15
**Testing dataset: GE Discovery MR 750w 3.0 T (36-channel body anterior coil)**									
T2-weighted 2D FSE	Yes	7826.1	98	142	6.5	7.5	320×320	380×380	148
Diffusion-weighted imaging*	Yes	8571.4	72.1	90	6	7.0	96×128	380×380	133
In- and out-of-phase T1-weighted imaging	No	5.3	2.3/1.1	12	4	2.0	288×180	390×390	14
Dynamic T1-weighted 3D GRE	Yes	5.4	2.0	12	5	2.5	288×160	380×380	15

Note. —TR = repetition time; TE = echo time; ST = section thickness; FOV = field of view; 2D = two-dimensional; 3D = three-dimensional; FSE = fast spin-echo; GRE = gradient recall echo. * Images were acquired under free breath.

Sequence	Fat suppression	TR (ms)	TE (ms)	Flip angle (°)	ST (mm)	Spacing (mm)	Matrix size	FOV (mm^2)	Acquisition Time (s)
**Testing dataset: Philips Achieva 3.0 T (16-channel body anterior coil)**									
T2-weighted 2D FSE	Yes	694.8	70.0	90	6	7	272×211	360×360	145
Diffusion-weighted imaging*	Yes	3000	54.7	90	7	8	132×135	380×380	172
In- and out-of-phase T1-weighted imaging	No	177.2	1.9/1.2	55	7	8	164×180	380×380	36
Dynamic T1-weighted 3D GRE	Yes	3	1.4	10	4	2	216×216	420×420	16
**Testing dataset: Philips Ingenia 3.0 T（Multi coil）**									
T2-weighted 2D FSE	Yes	2725	77.6	90	5	6	296×58	432×432	126
Diffusion-weighted imaging*	Yes	1405.1	55.2	90	5	6	120×116	384×384	168
In- and out-of-phase T1-weighted imaging	No	3.6	2.4/1.2	10	4	2	252×248	448×448	24
Dynamic T1-weighted 3D GRE	Yes	3.8	0	10	4	2	268×266	480×480	14

Note. —TR = repetition time; TE = echo time; ST = section thickness; FOV = field of view; 2D = two-dimensional; 3D = three-dimensional; FSE = fast spin-echo; GRE = gradient recall echo. * Images were acquired under free breath.

Sequence	Fat suppression	TR (ms)	TE (ms)	Flip angle (°)	ST (mm)	Spacing (mm)	Matrix size	FOV (mm^2)	Acquisition Time (s)
**Testing dataset: Siemens Verio 3.0 T (Tim coil)**									
T2-weighted 2D FSE	Yes	1800	98	160	6	7.8	320×120	172×320	130
Diffusion-weighted imaging*	Yes	1500	73	90	6	7.8	128×78	96×128	128
In- and out-of-phase T1-weighted imaging	No	200	2.9/1.5	70	6	7.8	256×126	210×320	22
Dynamic T1-weighted 3D GRE	Yes	3.9	1.4	9	3	-	320×147	210×320	15
**Testing dataset: Siemens MAGNETOM Vida 3.0 T (18-channel body array coil)**									
T2-weighted 2D FSE	Yes	2500	91	160	5	14	256×256	480×640	121
Diffusion-weighted imaging*	Yes	5600	44	90	6	7.5	134×108	216×268	137
In- and out-of-phase T1-weighted imaging	No	4.0	2.5/1.3	9	3	-	320×195	260×320	14
Dynamic T1-weighted 3D GRE	Yes	3.3	1.2	15	3	-	352×232	290×352	15

Note. —TR = repetition time; TE = echo time; ST = section thickness; FOV = field of view; 2D = two-dimensional; 3D = three-dimensional; FSE = fast spin-echo; GRE = gradient recall echo. * Images were acquired under free breath.

Sequence	Fat suppression	TR (ms)	TE (ms)	Flip angle (°)	ST (mm)	Spacing (mm)	Matrix size	FOV (mm^2)	Acquisition Time (s)
**Testing dataset: uMR780 3.0 T (12-channel body anterior coil)**									
T2-weighted 2D FSE	Yes	2140	103.3	100	6.5	1.3	256×256	380×300	228
Diffusion-weighted imaging*	Yes	4000	69.4	90	6.5	1.3	128×101	380×300	147
In- and out-of-phase T1-weighted imaging	No	148	2.9/1.5	70	6.5	1.3	272×174	400×320	32
Dynamic T1-weighted 3D GRE	Yes	3.4	1.6	10	4.5	1.5	320×204	400×300	15

Note. —TR = repetition time; TE = echo time; ST = section thickness; FOV = field of view; 2D = two-dimensional; 3D = three-dimensional; FSE = fast spin-echo; GRE = gradient recall echo. * Images were acquired under free breath.

Supplemental Table 2: Definitions of the imaging features.

Imaging features	Definition	Image sequence
LI-RADS major features		
Nonrim arterial phase hyperenhancement (present vs. absent)	Nonrim-like enhancement of the tumor in the arterial phase unequivocally greater in whole or in part than the liver. (1,2)	Late arterial phase
Nonperipheral washout (present vs. absent)	Nonperipheral visually assessed temporal reduction in the enhancement of the tumor in whole or in part relative to composite liver tissue in the portal venous phase or delayed phase. (1,2)	Portal venous or delayed phase for extracellular contrast agents or gadobenate; portal venous phase for gadoxetate
Enhancing capsule (present vs. absent)	Smooth, uniform, sharp border around most or all of a tumor, unequivocally thicker or more conspicuous than fibrotic tissue around background nodules, and visible as enhancing rim in portal venous phase, delayed phase, or transitional phase. (1,2)	Portal venous, delayed, or transitional phase
Tumor size (cm)	Largest outer-edge-to-outer-edge dimension of a tumor. (1,2)	Pick the phase, sequence, plane in which the margins are clearest. Do not measure in arterial phase or DWI.
LI-RADS ancillary features		
Diffusion restriction (present vs. absent)	Signal intensity of the tumor higher than the liver on diffusion-weighted images not caused only by T2 shine-through. (1,2)	High b value (e.g.,≥800) DWI
Mild-moderate T2 hyperintensity (present vs. absent)	Signal intensity of the tumor on T2WI higher than the liver, similar to or lower than a non-iron-overloaded spleen, and lower than simple fluid. (1,2)	T2WI
Corona enhancement (present vs. absent)	Peritumoral enhancement in the late arterial phase or early portal venous phase. The enhancement is contiguous with and surrounds all or part of the tumor. (1,2)	Late arterial or early portal venous phase
Nonenhancing capsule (present vs. absent)	Subtype of capsule that does not show enhancement on any image. (1,2)	Multiple sequences
Nodule-in-nodule architecture (present vs. absent)	Presence of smaller inner nodule within and having different imaging features than larger outer nodule. (1,2)	Multiple sequences
Mosaic architecture (present vs. absent)	Presence of any combination of internal nodules, compartments, or septations, within a solid or mostly solid mass. (1,2)	Multiple sequences
Blood products in mass (present vs. absent)	Intralesional or perilesional hemorrhage in the absence of biopsy, trauma, or intervention. (1,2)	Multiple sequences
Fat in mass, more than adjacent liver (present vs. absent)	Excess fat within a mass, in whole or in part, relative to the adjacent liver. (1,2)	In-/opposed phase; fat-suppressed images compared to non-fat-suppressed images with similar or identical weighting
Fat sparing in solid mass (present vs. absent)	Relative paucity of fat in solid mass relative to steatotic liver OR in inner nodule relative to steatotic outer nodule. (1,2)	In-/opposed phase; fat-suppressed images compared to non-fat-suppressed images with similar or identical weighting

Iron sparing in solid mass<br>(present vs. absent)	Paucity of iron in solid mass relative to iron-overloaded liver OR in inner nodule relative to siderotic outer nodule. (1,2)	In-/opposed phase; T2WI
Transitional phase hypointensity<br>(preset vs. absent)	Signal intensity of the tumor in the transitional phase unequivocally less, in whole or in part, than the liver. (1,2)	Transitional phase
Hepatobiliary phase hypointensity<br>(preset vs. absent)	Signal intensity of the tumor in the hepatobiliary phase unequivocally less, in whole or in part, than the liver. (1,2)	Hepatobiliary phase
Marked T2 hyperintensity<br>(present vs. absent)	Signal intensity of the tumor on T2WI higher than non-iron-overloaded spleen and as high as or almost as high as simple fluid. (1,2)	T2WI
Iron in mass, more than liver<br>(present vs. absent)	Excess iron in a mass relative to the background liver. (1,2)	In-/opposed phase; T2WI
Parallels blood pool enhancement<br>(present vs. absent)	Temporal pattern in which enhancement approximates blood pool in all phases. (1,2)	Post-contrast phases
Undistorted vessels<br>(present vs. absent)	Vessels traversing a tumor without displacement, deformation, or other alteration. (1,2)	Post-contrast phases
LR-M features		
Rim arterial phase hyperenhancement<br>(present vs. absent)	Spatially defined subtype of arterial phase hyperenhancement in which arterial phase enhancement is most pronounced in tumor periphery. (1,2)	Late arterial phase
Peripheral washout<br>(present vs. absent)	Presence of apparent washout most pronounced in the tumor periphery. (1,2)	Portal venous or delayed phase for extracellular contrast agents or gadobenate; portal venous phase for gadoxetate
Delayed central enhancement<br>(present vs. absent)	Central area of progressive postarterial phase enhancement. (1,2)	Postarterial phases
Targetoid restriction<br>(present vs. absent)	Concentric pattern on diffusion-weighted imaging characterized by restricted diffusion in tumor periphery with less restricted diffusion in tumor center. (1,2)	DWI or ADC map
Targetoid transitional phase or hepatobiliary phase appearance<br>(present vs. absent)	Concentric pattern in transitional or hepatobiliary phase characterized by moderate-to-marked hypointensity in the tumor periphery with milder hypointensity in the center. (1,2)	Transitional or hepatobiliary phase
Marked diffusion restriction<br>(present vs. absent)	Signal intensity of the tumor similar to or higher than non-iron-overloaded spleen on diffusion-weighted images not caused only by T2 shine-through. (1,2)	High b value (e.g.,≥800) DWI
Infiltrative appearance<br>(present vs. absent)	Presence of a non-circumscribed tumor margin (indistinct transition) thought to represent a permeative growth pattern. (1,2)	Multiple sequences
Necrosis or severe ischemia<br>(present vs. absent)	Presence of nonenhancing area in a solid mass, not attributable to a cystic component, prior treatment, or intralesional hemorrhage. (1,2)	Post-contrast phases and T2WI
Other tumor-related prognostic features		

Pre-contrast T1-weighted hypointensity (present vs. absent)	Signal intensity of the tumor on pre-contrast T1-weighted imaging unequivocally lower than that of the liver.	Pre-contrast T1WI
T2-weighted peritumoral hyperintensity (present vs. absent)	Presence of irregular, wedge-shaped, or flame-like area adjacent to the tumor on T2WI of which the signal intensity is mildly or moderately higher than liver and similar to or less than a non-iron-overloaded spleen. (3,4)	T2WI
Portal venous phase peritumoral hypoenhancement (present vs. absent)	Presence of irregular, wedge-shaped, or flame-like hypoenhancing area adjacent to the tumor in the portal venous phase. (3,4)	Portal venous phase
Arterial phase hyperenhancement proportion (<50% vs. ≥50%)	Proportion of tumor volume demonstrating arterial phase hyperenhancement. (5)	Late arterial phase
Intratumoral artery (present vs. absent)	Presence of discrete arteries within the tumor on arterial phase images. (3,6,7)	Early or late arterial phase
Capsule integrity (complete vs. incomplete)	Tumor capsule is complete when non-disrupted capsule is detected in all imaging planes, otherwise incomplete. (3,8)	Multiple sequences
Tumor margin (nonsmooth vs. smooth)	Nonsmooth: the tumor margin is irregular and/or has areas of bulging, nodular projection, or infiltration into adjacent tissues at the tumor periphery in any imaging plane.<br>Smooth: the tumor margin is smooth in all imaging planes. (3,7,9)	Multiple sequences
Marked hepatobiliary phase hypointensity (present vs. absent)	Signal intensity of the tumor in the hepatobiliary phase lower than that of the liver and similar to or lower than that of intrahepatic vessels. (10)	Hepatobiliary phase
Hepatobiliary phase peritumoral hypointensity (present vs. absent)	Presence of irregular, wedge-shaped or flame-like hypointense area of liver parenchyma located outside of the tumor margin in the hepatobiliary phase. (3,4,10,11)	Hepatobiliary phase
The VICT2 trait (present vs. absent)	The VICT2 trait is present when portal venous phase peritumoral hypoenhancement is present; or when corona enhancement, T2-weighted peritumoral mild-moderate hyperintensity, and incomplete capsule are all present; otherwise, absent. (4)	Multiple sequences
The two-trait predictor of venous invasion (present vs. absent)	Presence of intratumoral artery coupled with absence of nonenhancing "capsule". (6,7)	Multiple sequences
Tumor growth subtype (12,13)		Multiple sequences
Simple nodular type	A round expanding nodule with a distinct margin in all imaging planes.	
Non-simple nodular type	An expanding tumor with areas of bulging or nodular extranodular projection, a cluster of small and confluent nodules, or the presence of an indistinct margin thought the entire tumor.	
Imaging features associated with the severity of underlying liver diseases and portal hypertension		
Ascites (present vs. absent)	Presence of free fluid in abdomen or pelvis. (14)	T2WI

Radiologically-evident cirrhosis (present vs. absent)	Unequivocal morphological alterations of the liver, including surface nodularity, small liver volume, expansion of space between liver and anterior abdominal wall and perihilar, gallbladder fossa and ligamentum teres spaces, hypertrophy of caudate and/or left lateral section, atrophy of anterior right section and/or left medial section, anterolateral flattening, notching of posterior medial right lobe and parenchymal nodules, with or without manifestations of portal hypertension (portal-systemic collaterals, splenomegaly and/or ascites). (1,2)	Multiple sequences
Diffuse iron overload (present vs. absent)	Diffuse signal intensity drop of the liver parenchyma on in-phase images compared with the out-of-phase images. (1,2)	In-/opposed phase; T2WI
Diffuse fatty change (present vs. absent)	Diffuse signal intensity drop of the liver parenchyma on out-of-phase images compared with the in-phase images. (1,2)	In-/opposed phase; fat-suppressed images compared to non-fat-suppressed images with similar or identical weighting
Width of main portal vein (cm)	Diameter of main portal vein, which is measured at least 1cm distal to the confluence of splenic and superior mesenteric vein and at least 1cm proximal to the first branch of the main portal vein, to avoid effect of convergence/divergence on coronal images. (15)	Coronal images of the portal venous or delayed phase
Splenomegaly (present vs. absent)	Craniocaudal diameter of the spleen>12cm. (14,16,17)	Multiple sequences
Porto-systemic shunts (present vs. absent)	Enhancing tortuous channels in esophageal, epigastric, perisplenic, paraumbilical or retroperitoneal locations. (14)	Portal venous or delayed phase
Esophageal gastric varices (present vs. absent)	Discrete enhancing tortuous channel abutting the luminal surface of the esophageal or gastric wall or contacting/ protruding into luminal space. (14)	Portal venous or delayed phase

References:

1. CT/MRI Liver Imaging Reporting and Data System version 2018. American College of Radiology Web site. https://www.acr.org/Clinical-Resources/Reporting-andData- Systems/LI-RADS/CTMRI-LI-RADS-v2018. Accessed 1 September 2021.
2. LI-RADS Lexicon (terms and definitions). American College of Radiology Web site. https://www.acr.org/-/media/ACR/Files/RADS/LI-RADS/LIRADS-Lexicon-Table.pdf. Accessed 1 September 2021.
3. Jiang H, Wei J, Fu F, et al. Predicting microvascular invasion in hepatocellular carcinoma: a dual-institution study on gadoxetate disodium-enhanced MRI. Liver Int. 2022;42(5):1158-1172.
4. Jiang H, Wei H, Yang T, et al. VICT2 Trait: Prognostic Alternative to Peritumoral Hepatobiliary Phase Hypointensity in HCC. Radiology. 2023 Feb 14:221835.
5. Rhee H, Cho ES, Nahm JH, et al. Gadoxetic acid-enhanced MRI of macrotrabecular-massive hepatocellular carcinoma and its prognostic implications. J Hepatol. 2021;74(1):109-121.
6. Segal E, Sirlin CB, Ooi C, Adler AS, Gollub J, Chen X, et al. Decoding global gene expression programs in liver cancer by noninvasive imaging. Nat Biotechnol 2007; 25:675–680.
7. Renzulli M, Brocchi S, Cucchetti A, Mazzotti F, Mosconi C Sportoletti C, et al. Can Current Preoperative Imaging Be Used to Detect Microvascular Invasion of Hepatocellular Carcinoma? Radiology 2016;279(2):432-442.
8. Lei Z, Li J, Wu D, Xia Y, Wang Q, Si A, et al. Nomogram for Preoperative Estimation of Microvascular Invasion Risk in Hepatitis B Virus-Related Hepatocellular Carcinoma Within the Milan Criteria. JAMA 2016;151(4):356-363.
9. An C, Kim DW, Park YN, Chung YE, Rhee H, Kim MJ. Single hepatocellular carcinoma: preoperative MR imaging to predict early recurrence after curative resection. Radiology 2015; 276:433–443.

10. Fowler KJ, Chernyak V, Ronot M, et al. Hepatocellular Carcinoma: It Is Time to Focus on Prognosis. Radiology. 2023:220884.
11. Lee S, Kim SH, Lee JE, Sinn DH, Preoperative gadoxetic acid-enhanced MRI for predicting microvascular invasion in patients with single hepatocellular carcinoma. J Hepatol 2017;67(3):526-534.
12. Rhee H, Chung T, Yoo JE, et al. Gross type of hepatocellular carcinoma reflects the tumor hypoxia, fibrosis, and stemness-related marker expression. Hepatol Int. 2020;14(2):239-248.
13. Burt AD, Alves V, Bedossa P, et al. Data set for the reporting of intrahepatic cholangiocarcinoma, perihilar cholangiocarci noma and hepatocellular carcinoma: recommendations from the International Collaboration on Cancer Reporting (ICCR). Histopathology. 2018;73:369–85.
14. Venkatesh SK, Yin M, Takahashi N, et al. Non-invasive detection of liver fibrosis: MR imaging features vs. MR elastography. Abdom Imaging. 2015;40(4):766-75.
15. Stamm ER, Meier JM, Pokharel SS, et al. Normal main portal vein diameter measured on CT is larger than the widely referenced upper limit of 13 mm. Abdom Radiol (NY). 2016;41(10):1931-6.
16. Rustogi R, Horowitz J, Harmath C, et al. Accuracy of MR elastography and anatomic MR imaging features in the diagnosis of severe hepatic fibrosis and cirrhosis. J Magn Reson Imaging. 2012;35(6):1356-64.
17. Marasco G, Colecchia A, Colli A, et al. Role of liver and spleen stiffness in predicting the recurrence of hepatocellular carcinoma after resection. J Hepatol. 2019;70(3):440-448.

Supplemental Table 3. Univariable logistic analyses of all non-invasive predictors for the high-risk histopathology based on majority interpretations of the training dataset.

Characteristics	Univariable logistic analysis	
	Odds ratio	*P* value
**Clinical and laboratory variables**		
Age (y)	0.99 (0.92-1.01)	*0.38*
Sex (male vs. female)	0.62 (0.35-1.09)	*0.10*
Chronic hepatitis B (yes vs. no)	1.13 (0.26-4.79)	*0.87*
The Barcelona Clinic Liver Cancer stage (A vs. 0)	1.79 (1.09-2.94)	***0.02***
Cirrhosis (yes vs. no)	0.99 (0.63-1.56)	*0.99*
The Child-Pugh score	0.79 (0.37-1.69)	*0.54*
The Child-Pugh class (B vs. A)	0.49 (0.05-4.77)	*0.54*
The albumin-bilirubin score	1.36 (0.78-2.37)	*0.28*
The albumin-bilirubin grade (2 vs. 1)	1.48 (0.84-2.62)	*0.17*
Alpha-fetoprotein (>100 ng/mL vs. ≤100 ng/mL)	1.98 (1.27-3.11)	***0.003***
Aspartate aminotransferase (IU/L)	1.00 (0.99-1.01)	*0.48*
Aspartate aminotransferase (IU/L)*	1.05 (0.63-1.74)	*0.86*
Aspartate aminotransferase (>35 IU/L vs. ≤35 IU/L)	1.09 (0.67-1.75)	*0.74*
Alanine aminotransferase (IU/L)	1.00 (0.99-1.01)	*0.77*
Alanine aminotransferase (IU/L)*	1.03 (0.72-1.49)	*0.86*
Alanine aminotransferase (>40 IU/L vs. ≤40 IU/L)	1.05 (0.65-1.68)	*0.85*
Total bilirubin (umol/L)	1.00 (0.96-1.04)	*0.96*
Total bilirubin (umol/L)*	1.26 (0.35-4.55)	*0.72*
Total bilirubin (>40 umol/L vs. ≤40 umol/L)	...	...
Albumin (g/L)	0.97 (0.93-1.02)	*0.30*
Albumin (g/L)*	1.51 (0.33-6.98)	*0.60*
Albumin (<40 g/L vs. ≥40 g/L)	1.29 (0.75-2.23)	*0.36*
Platelet count (^9/L)	1.00 (1.00-1.01)	*0.49*
Platelet count (^9/L)*	1.12 (0.72-1.75)	*0.62*
Platelet count (<100^9/L vs. ≥100^9/L)	1.19 (0.77-1.86)	*0.43*
Neutrophil count (^9/L)	1.00 (0.89-1.14)	*0.95*
Neutrophil count (^9/L)*	1.07 (0.65-1.77)	*0.78*
Neutrophil count (≤6.3×10^9/L vs.>6.3×10^9/L)	1.50 (0.43-5.30)	*0.53*
Lymphocyte count (^9/L)	0.97 (0.70-1.35)	*0.87*
Lymphocyte count (^9/L)*	1.04 (0.64-1.70)	*0.88*
The neutrophil-to-lymphocyte ratio	1.01 (0.95-1.08)	*0.73*
Prothrombin time (s)	1.03 (0.85-1.26)	*0.73*
Prothrombin time (s)*	1.65 (0.15-18.02)	*0.68*
Prothrombin time (>12.8 s vs. ≤12.8 s)	0.92 (0.50-1.68)	*0.78*
International normalized ratio	0.91 (0.11-7.75)	*0.93*
International normalized ratio*	0.99 (0.10-9.53)	*>0.99*
International normalized ratio (>1.15 vs. ≤1.15)	0.85 (0.42-1.71)	*0.65*
**MR imaging variables**		
LI-RADS major features		

Nonrim arterial phase hyperenhancement (present vs. absent)	0.65 (0.29-1.45)	*0.29*
Nonperipheral washout (present vs. absent)	1.60 (0.97-2.63)	*0.07*
Enhancing capsule (present vs. absent)	1.55 (0.68-3.51)	*0.29*
Tumor size (cm)	1.31 (1.06-1.62)	***0.01***
LI-RADS ancillary features		
Corona enhancement (present vs. absent)	2.23 (1.39-3.57)	***<0.001***
Mosaic architecture (present vs. absent)	1.44 (0.69-3.02)	*0.33*
Blood products in mass (present vs. absent)	1.19 (0.58-2.44)	*0.63*
Diffusion restriction (present vs. absent)	...	...
Mild-moderate T2 hyperintensity (present vs. absent)	1.01 (0.35-2.91)	*0.99*
Nonenhancing capsule (present vs. absent)	0.99 (0.16-6.00)	*0.99*
Fat in mass, more than adjacent liver (present vs. absent)	0.84 (0.53-1.32)	*0.45*
Fat sparing in solid mass (present vs. absent)	0.79 (0.31-2.03)	*0.62*
Iron sparing in solid mass (present vs. absent)	0.59 (0.34-1.01)	*0.054*
Transitional phase hypointensity (preset vs. absent)†	1.11 (0.27-4.62)	*0.89*
Hepatobiliary phase hypointensity (preset vs. absent)†	2.85 (0.32-25.04)	*0.35*
Marked T2 hyperintensity (present vs. absent)	1.82 (0.54-6.08)	*0.33*
Nodule-in-nodule architecture (present vs. absent)	1.34 (0.82-2.18)	*0.25*
Iron in mass, more than liver (present vs. absent)	...	...
Parallels blood pool enhancement (present vs. absent)	...	...
Undistorted vessels (present vs. absent)	...	...
LR-M features		
Rim arterial phase hyperenhancement (present vs. absent)	2.35 (0.93-5.90)	*0.07*
Peripheral washout (present vs. absent)	1.49 (0.09-24.01)	*0.78*
Delayed central enhancement (present vs. absent)	0.29 (0.03-2.53)	*0.26*
Targetoid diffusion restriction (present vs. absent)	...	...
Targetoid TP or HBP appearance (present vs. absent)†	1.83 (0.11-29.86)	*0.67*
Marked diffusion restriction (present vs. absent)	0.72 (0.35-1.45)	*0.72*
Infiltrative appearance (present vs. absent)	3.82 (0.73-19.96)	*0.11*
Necrosis or severe ischemia (present vs. absent)	2.39 (1.33-4.30)	***0.004***
LI-RADS category		
LR-5	*Ref*	...
LR-4	0.63 (0.32-1.27)	*0.20*
LR-M	2.09 (0.77-5.64)	*0.15*
Other tumor-related prognostic features		
Precontrast T1-weighted hypointensity (yes vs. no)	1.86 (0.71-4.88)	*0.21*
Peritumoral mild-moderate T2 hypointensity (present vs. absent)	3.79 (1.93-7.46)	***<0.001***
Portal venous phase peritumoral hypoenhancement (present vs. absent)	3.71 (1.75-7.86)	***<0.001***
Arterial phase hyperenhancement proportion (<50% vs. ≥50%)	1.15 (0.70-1.87)	*0.58*
Intratumoral arteries (present vs. absent)	1.41 (0.74-2.70)	*0.30*
Capsule integrity (complete vs. incomplete)	0.50 (0.31-0.79)	***0.003***
Tumor margin (nonsmooth vs. smooth)	1.83 (1.15-2.91)	***0.01***
Marked HBP hypointensity (present vs. absent)†	2.17 (1.09-4.33)	***0.03***
HBP peritumoral hypointensity (present vs. absent)†	2.16 (1.04-4.49)	***0.04***
The VICT2 trait (present vs. absent)	5.18 (2.81-9.55)	***<0.001***
The two-trait predictor of venous invasion (present vs. absent)	1.41 (0.74-2.70)	*0.30*

Tumor growth subtype (non-simple nodular type vs. simple nodular type)	2.11 (1.36-3.28)	***<0.001***
Involvement of liver capsule (yes vs. no)	0.86 (0.56-1.33)	*0.51*
Imaging features associated with the severity of underlying liver diseases and portal hypertension		
Ascites (present vs. absent)	0.33 (0.09-1.17)	*0.09*
Radiologically-evident cirrhosis (present vs. absent)	1.17 (0.71-1.92)	*0.54*
Diffuse iron overload (present vs. absent)	0.71 (0.44-1.15)	*0.16*
Diffuse fatty change (present vs. absent)	1.35 (0.72-2.54)	*0.35*
Width of main portal vein (cm)	0.46 (0.17-1.26)	*0.13*
Splenomegaly (present vs. absent)	1.07 (0.71-1.62)	*0.75*
Porto-systemic shunts (present vs. absent)	1.24 (0.79-1.94)	*0.35*
Esophageal gastric varices (present vs. absent)	1.22 (0.79-1.88)	*0.37*

Note.—Unless stated otherwise, data in parentheses are 95% confidence intervals.

*Data are computed after logarithmic transformation.

†Data are presented for training dataset patients who underwent hepatobiliary contrast agent-enhanced MRI (n=152).

LI-RADS = Liver Imaging Reporting and Data System; TP = transitional phase; HBP = hepatobiliary phase.

Supplemental Table 4. Frequencies and inter-rater agreement for all analysed MR imaging features.

Imaging features	Frequency			κ value or ICC*			Agreement		
	Training dataset (*n*=343)	Testing dataset (*n*=113)	The RFA cohort (*n*=121)	Training dataset (*n*=343)	Testing dataset (*n*=113)	The RFA cohort (*n*=121)	Training dataset (*n*=343)	Testing dataset (*n*=113)	The RFA cohort (*n*=121)
Tumor size (cm)	...	...	...	0.982 (0.977-0.986)	0.992 (0.989-0.994)	0.979 (0.971-0.984)	Excellent	Excellent	Excellent
Corona enhancement (present vs. absent)	102 (29.7)	45 (39.8)	28 (23.1)	0.304 (0.221-0.386)	0.468 (0.349-0.588)	0.457 (0.354-0.559)	Fair	Moderate	Moderate
Peritumoral mild-moderate T2 hypointensity (present vs. absent)	44 (12.8)	23 (20.4)	25 (20.7)	0.468 (0.386-0.550)	0.485 (0.366-0.604)	0.452 (0.349-0.555)	Moderate	Moderate	Moderate
Portal venous phase peritumoral hypoenhancement (present vs. absent)	35 (10.2)	20 (17.7)	11 (9.1)	0.397 (0.315-0.479)	0.577 (0.458-0.696)	0.489 (0.387-0.592)	Fair	Moderate	Moderate
Capsule integrity (complete vs. incomplete)	124 (36.2)	35 (31.0)	64 (52.9)	0.396 (0.314-0.478)	0.447 (0.328-0.566)	0.389 (0.286-0.492)	Fair	Moderate	Fair
The VICT2 trait (present vs. absent)	61 (17.8)	32 (28.3)	18 (14.9)	0.439 (0.357-0.521)	0.494 (0.375-0.613)	0.512 (0.409-0.615)	Moderate	Moderate	Moderate
Tumor growth subtype (non-simple nodular type vs. simple nodular type)	171 (49.9)	74 (65.5)	40 (33.1)	0.367 (0.284-0.449)	0.499 (0.380-0.618)	0.586 (0.483-0.689)	Fair	Moderate	Moderate

Note.—Unless stated otherwise, data in parentheses are percentages or 95% confidence intervals.

* Interobserver agreement was investigated by computing the Fleiss' κ value for binary variables and the intraclass correlation coefficient (ICC) for tumor size. Agreement was considered poor (κ or ICC < 0.0), slight (κ or ICC: 0.2-0.4), fair (κ or ICC: 0.2-0.4), moderate (κ or ICC: 0.4-0.6), substantial (κ or ICC: 0.6-0.8), or excellent (κ or ICC > 0.8).

RFA = radiofrequency ablation; ICC = intraclass correlation coefficient; LI-RADS = Liver Imaging Reporting and Data System.

Supplemental Table 5. Uni- and multivariable Cox regression analyses of the MHG trait and known prognostic factors for recurrence-free survival.

Characteristics	Univariable Cox analysis		Multivariable Cox analysis*	
	Hazard ratio	*P value*	Hazard ratio	*P value*
**The resection cohort, training dataset (*n*=274)**				
The MHG trait (positive vs. negative)	1.86 (1.22-2.85)	***0.004***	1.61 (1.03-2.51)	***0.04***
Clinical prognostic factors				
Age (years)	0.99 (0.97-1.01)	*0.43*	...	...
Sex (male vs. female)	0.77 (0.43-1.40)	*0.39*	...	...
Chronic hepatitis B virus infection (yes vs. no)	1.87 (0.76-4.61)	*0.17*		
Cirrhosis (present vs. absent)	1.17 (0.75-1.82)	*0.50*	...	...
Tumor size (cm)	1.38 (1.13-1.68)	***0.001***	1.29 (1.05-1.58)	***0.02***
The Barcelona Clinic Liver Cancer stage (A vs. 0)	1.80 (1.08-2.99)	***0.02***	...	...
The albumin-bilirubin grade (2 vs. 1)	2.23 (1.35-3.69)	***0.002***	2.23 (1.35-3.69)	***0.002***
Aspartate aminotransferase (IU/L)	1.00 (0.99-1.01)	*0.81*	...	...
Alanine aminotransferase (IU/L)	1.00 (0.99-1.00)	*0.37*	...	...
Total bilirubin (umol/L)	1.00 (0.96-1.04)	*0.95*	...	...
Albumin (g/L)	0.97 (0.92-1.02)	*0.21*	...	...
Platelet count (^9/L)	1.00 (0.99-1.00)	*0.24*	...	...
Prothrombin time (s)	1.09 (0.89-1.33)	*0.42*	...	...
Treatment-associated prognostic factors				
Adjuvant therapy (yes vs. no)	0.94 (0.59-1.50)	*0.79*	...	...
**The resection cohort, testing dataset (*n*=85)**				
The MHG trait (positive vs. negative)	4.02 (1.42-11.37)	***0.009***	3.55 (1.25-10.10)	***0.02***
Clinical prognostic factors				
Age (years)	0.99 (0.95-1.04)	*0.77*	...	...
Sex (male vs. female)	…	*0.95*	...	...
Chronic hepatitis B virus infection (yes vs. no)	0.87 (0.20-3.82)	*0.86*		
Cirrhosis (present vs. absent)	1.06 (0.41-2.77)	*0.91*	...	...
Tumor size (cm)	1.68 (1.14-2.46)	***0.009***	1.57 (1.08-2.26)	***0.02***
The Barcelona Clinic Liver Cancer stage (A vs. 0)	…	*0.96*	...	...
The albumin-bilirubin grade (2 vs. 1)	0.63 (0.22-1.78)	*0.38*	...	...
Aspartate aminotransferase (IU/L)	1.00 (0.99-1.01)	*0.61*	...	...
Alanine aminotransferase (IU/L)	1.00 (1.00-1.01)	*0.40*	...	...
Total bilirubin (umol/L)	1.02 (0.97-1.09)	*0.41*	...	...
Albumin (g/L)	1.06 (0.96-1.17)	*0.23*	...	...
Platelet count (^9/L)	1.00 (0.99-1.01)	*0.62*	...	...
Prothrombin time (s)	0.97 (0.72-1.30)	*0.82*	...	...
Treatment-associated prognostic factors				
Adjuvant therapy (yes vs. no)	0.89 (0.32-2.49)	*0.82*	...	...
**The RFA cohort (*n*=121)**				
The MHG trait (positive vs. negative)	3.20 (1.92-5.35)	***<0.001***	3.45 (2.01-5.80)	***<0.001***
The IMBRAVE 050-defined high-risk status (present vs. absent)†	2.01 (1.21-3.33)	***0.007***	…	…
Clinical prognostic factors				
Age (years)	1.01 (0.99-1.03)	*0.37*	...	...
Sex (male vs. female)	2.23 (1.02-4.88)	***0.046***	...	...
Chronic hepatitis B virus infection (yes vs. no)	0.53 (0.29-0.97)	***0.04***	0.46 (0.25-0.84)	***0.01***
Cirrhosis (present vs. absent)	0.99 (0.57-1.72)	*0.97*	...	...
Tumor size (cm)	1.64 (1.22-2.20)	***<0.001***	...	...
The Barcelona Clinic Liver Cancer stage (A vs. 0)	2.01 (1.21-3.33)	***0.007***	...	...
The albumin-bilirubin grade (2/3 vs. 1)	1.02 (0.63-1.66)	*0.94*		
Aspartate aminotransferase (IU/L)	1.01 (0.996-1.02)	*0.19*	...	...
Alanine aminotransferase (IU/L)	1.01 (1.00-1.02)	*0.13*	...	...
Total bilirubin (umol/L)	1.00 (0.97-1.02)	*0.70*	...	...
Albumin (g/L)	1.00 (0.95-1.05)	*0.98*	...	...
Platelet count (^9/L)	1.00 (1.00-1.01)	*0.90*	...	...
Prothrombin time (s)	0.99 (0.83-1.19)	*0.95*	...	...
Treatment-associated prognostic factors				
Adjuvant therapy (yes vs. no)	0.63 (0.30-1.33)	*0.23*	...	...

Note.—Unless stated otherwise, data in parentheses are 95% confidence intervals. *P* values<0.05 are highlighted in bold.

*To avoid overfitting, variables with *P*<0.05 at the univariable Cox regression analyses were analyzed in the multivariable Cox regression models.

†The IMBRAVE 050-defined high-risk status was single tumor >2 cm but ≤5 cm (7).

MHG = the microvascular invasion or high-grade trait; RFA = radiofrequency ablation.

Supplemental Table 6. Details of adjuvant therapies.

Adjuvant therapy regimens	No. of patients
The resection cohort (*n*=100)	
transarterial chemoembolization (once, performed within 3 months after surgery)	55 (55.0)
transarterial chemoembolization (once, performed within 3 months after surgery) + apatinib (750 mg, oral, once a day; started within 3 months after surgery and treated until recurrence or dose-limiting toxicity)	1 (1.0)
transarterial chemoembolization (once, performed within 3 months after surgery) + sorafenib (oral; 200 mg, twice a day; or 200 mg, once a day; or 400 mg, twice a day; started within 3 months after surgery and treated until recurrence or dose-limiting toxicity)	11 (11.0)
transarterial chemoembolization (once, performed within 3 months after surgery) + pembrolizumab (200 mg, intravenous; every three weeks; started within 3 months after surgery and treated until recurrence or dose-limiting toxicity)	2 (2.0)
transarterial chemoembolization (once, performed within 3 months after surgery) + camrelizumab (200 mg, intravenous; every three weeks; started within 3 months after surgery and treated until recurrence or dose-limiting toxicity) + lenvatinib (8 mg, oral, once a day; started within 3 months after surgery and treated until recurrence or dose-limiting toxicity)	3 (3.0)
radiation therapy (for the marginal parenchyma and the tumor bed; once, performed within 3 months after surgery)	1 (1.0)
sorafenib (oral; 200 mg, twice a day; or 200 mg, once a day; or 400 mg, twice a day; started within 3 months after surgery and treated until recurrence or dose-limiting toxicity)	12 (12.0)
lenvatinib (8 mg, oral, once a day; started within 3 months after surgery and treated until recurrence or dose-limiting toxicity)	6 (6.0)
atezolizumab (1200 mg, intravenous) + bevacizumab (15 mg/kg, intravenous) (started within 3 months after surgery and treated for 12 months, or recurrence, or dose-limiting toxicity, whichever occurred first)	3 (3.0)
pembrolizumab (200 mg, intravenous; every three weeks; started within 3 months after surgery and treated until recurrence or dose-limiting toxicity)	3 (3.0)

toripolimab (240 mg, intravenous; started within 3 months after surgery and treated for 12 months, or until recurrence or dose-limiting toxicity)	2 (2.0)
tegafur,gimeracil and oteracil porassium (20 mg, oral, once a day; started within 3 months after surgery and treated until 6 months)	1 (1.0)
The RFA cohort (*n*=17)	
transarterial chemoembolization (once, performed within 3 months after RFA)	10 (58.8)
Y90 radioembolization (for the RFA area and peripheral parenchyma; once, performed within 3 months after surgery)	1 (5.9)
sorafenib (oral; 200 mg, twice a day; or 200 mg, once a day; or 400 mg, twice a day; started within 3 months after surgery and treated until recurrence or dose-limiting toxicity)	2 (11.8)
lenvatinib (8 mg, oral, once a day; started within 3 months after surgery and treated until recurrence or dose-limiting toxicity)	3 (17.6)
atezolizumab (1200 mg, intravenous) + bevacizumab (15 mg/kg, intravenous) (started within 3 months after surgery and treated for 12 months, or recurrence, or dose-limiting toxicity, whichever occurred first)	1 (5.9)

Note.—Data in parentheses are percentages. RFA = radiofrequency ablation.

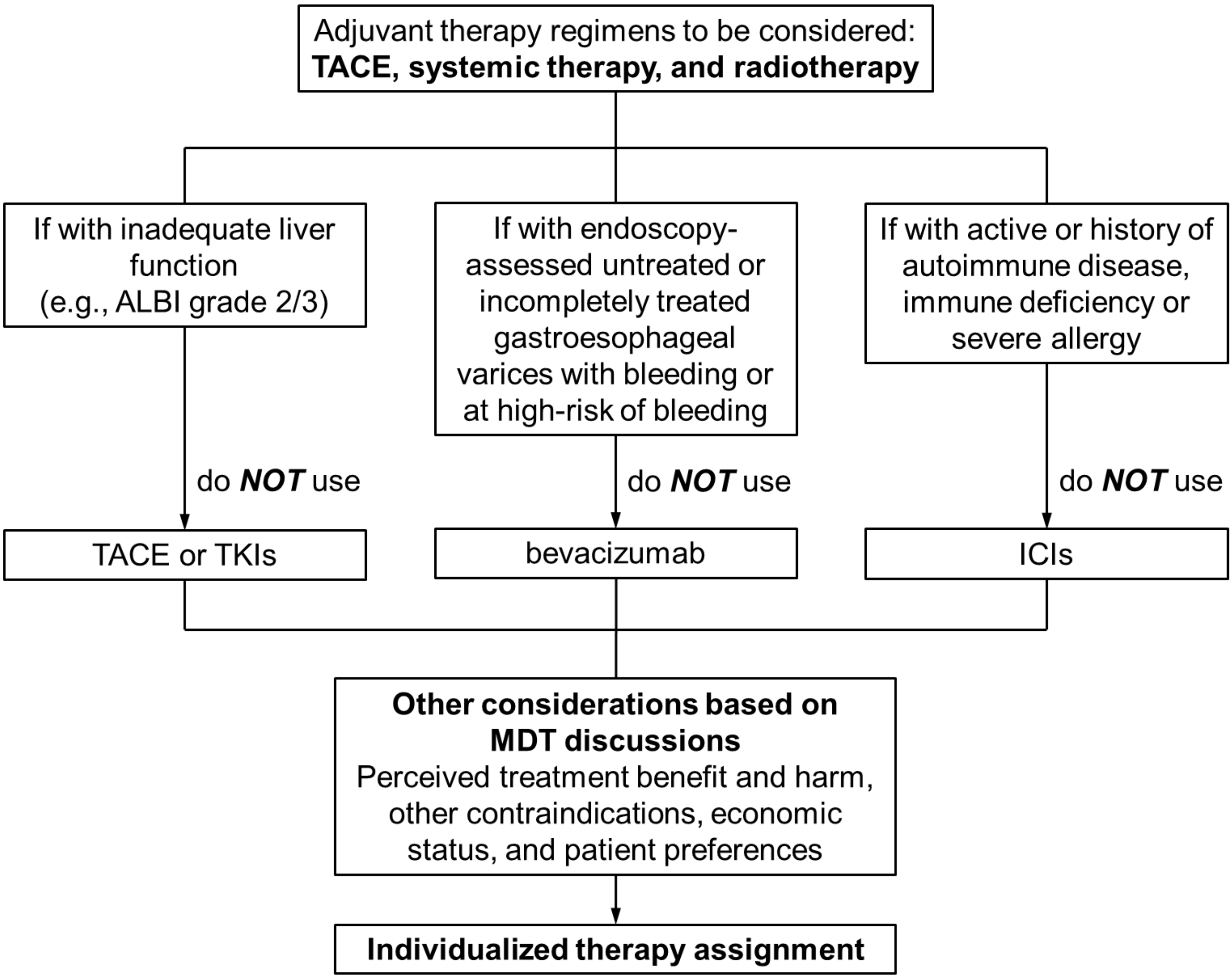

Supplemental Figure 1. Schematic for adjuvant therapy allocation.

Abbreviations: ICI, immune checkpoint inhibitor; MDT, multidisciplinary team; TACE, transarterial chemoembolization; TKI, tyrosine Kinase Inhibitors.

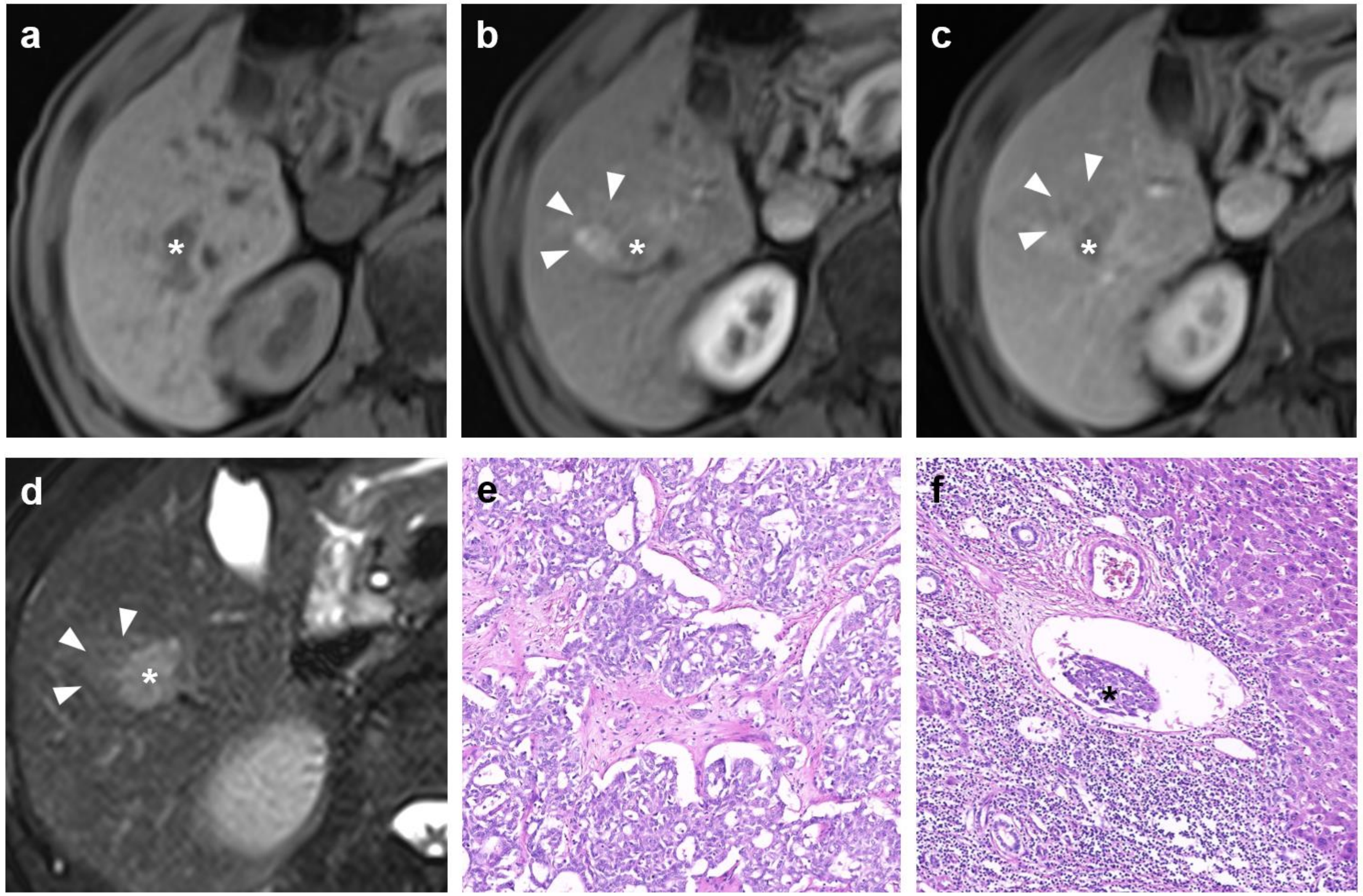

Supplemental Figure 2. Contrast-enhanced MR images of a 48-year-old male patient with chronic hepatitis B and a serum α-fetoprotein of >1210 ng/mL. A 2.7 cm hepatocellular carcinoma with non-simple nodular growth type (***white star***) was detected in segment V. The tumor shows hypointensity on T1-weighted pre-contrast images (**a**), nonrim hyperenhancement and corona enhancement (***arrowhead***) in the late arterial phase (**b**), and nonperipheral washout (***white star***) and peritumoral hypoenhancement (***arrowhead***) in the portal venous phase (**c**). The tumor (***white star***) also demonstrates mild-moderate hyperintensity on T2-weighted images (**d**) with peritumoral mild-to-moderate hyperintensity (***arrowhead***). Considering the presence of corona enhancement, portal venous phase peritumoral hypoenhancement, and peritumoral mild-moderate T2 hyperintensity, the VICT2 trait is present. This patient is thus assigned an MHG-positive status according to the MHG trait. The tumor was histopathologically confirmed as an Edmondson-Steiner G3 tumor (**e**; hematoxylin-eosin stain; magnification, ×100) with microvascular invasion (***black star*, f**; with hematoxylin-eosin stain; magnification, ×100). The patient experienced recurrence 121 days after curative resection.

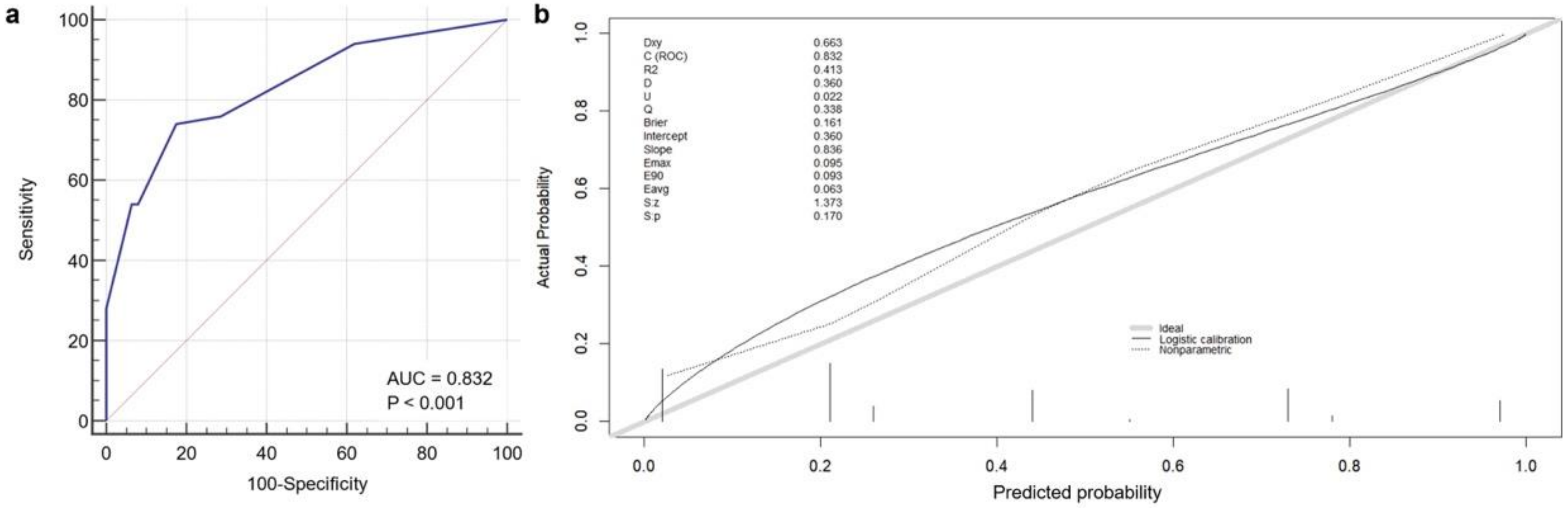

Supplemental Figure 3. Testing dataset receiver operating characteristic curve (***a***) and calibration curve (***b***) of the MVI or high-grade (MHG) score based on majority interpretations.
